# Tuning Linkers in Azo-Linked Porphyrin-Based Porous Organic Polymers for Enhanced CO_2_ Capture

**DOI:** 10.1021/acs.langmuir.5c05572

**Published:** 2026-02-01

**Authors:** Matija Popović, Tea Frey, Mladen Borovina, Ivan Kodrin, Ivana Biljan

**Affiliations:** 117036 University of Zagreb, Faculty of Science, Department of Chemistry, Horvatovac 102a, HR-10000 Zagreb, Croatia

## Abstract

The increased concentration of atmospheric CO_2_ and its impact on the environment drive the search for new materials that can capture and store this greenhouse gas effectively. Porous organic polymers (POPs) are very promising materials for this task because of their stability and the tunability of their pore structure and chemistry. In this study, we synthesized a series of azo-linked porphyrin-based porous organic polymers (APPs) using either heteroatom-containing linkers (hydroxylated biphenyl in APP-BP-OH and carbonyl-bearing anthraquinone in APP-AQ) or sterically hindered linkers (methylated biphenyl and phenyl in APP-BP-Me and APP-Ph-Me, respectively). Structural characterization confirmed the formation of azo linkages and the amorphous nature of the frameworks, while thermal analysis showed that APPs are stable up to at least 200 °C. Gas sorption studies revealed notable differences in porosity and CO_2_ uptake. APP-Ph-Me exhibited the largest surface area (673 m^2^ g^–1^), whereas APP-BP-OH had a smaller surface area (488 m^2^ g^–1^) but adsorbed more CO_2_ (49 mg g^–1^) compared to APP-Ph-Me (41 mg g^–1^). These results illustrate that CO_2_ adsorption in APPs is governed by not only the surface area but also the chemical environment. Nitrogen-rich porphyrin and azo moieties play a dominant role, while polar hydroxyl groups and biphenyl linkers provide additional contributions. Computational results supported these findings, indicating that hydroxyl and carbonyl groups create favorable binding sites, while methyl groups limit accessibility to porphyrin and azo regions. Overall, our results highlight how linker design and functionalization directly influence porosity and adsorption performance and offer useful guidelines for the development of new POPs for CO_2_ capture.

## Graphical abstract

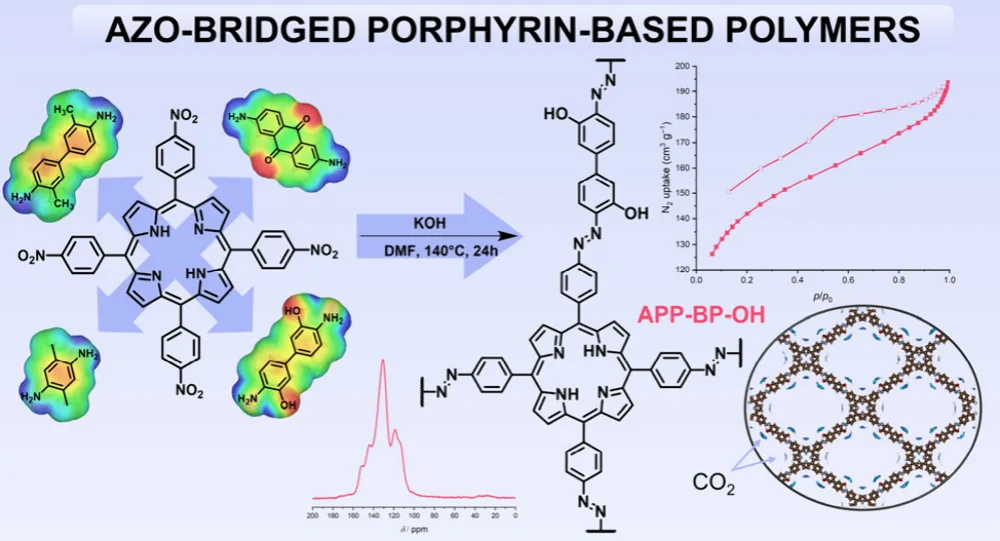

## Introduction

Climate change presents one of the most serious challenges today, with far-reaching consequences for the environment, society and economy. This phenomenon is driven by the rising concentration of greenhouse gases in the atmosphere, primarily carbon dioxide (CO_2_), which is largely emitted through the combustion of fossil fuels and other human activities.^1,2^ Prior to the industrial revolution, atmospheric CO_2_ levels were stable at around 280 ppm for thousands of years. Since then, human activities have raised atmospheric CO_2_ to over 425 ppm,^3^ which is more than 50% higher than preindustrial levels.^4^ In view of the above, the development of new materials for capturing and storing CO_2_ is crucial for mitigating climate change. Various porous materials, including zeolites, metal**-**organic frameworks (MOFs), activated carbons and porous organic polymers (POPs), have gained significant attention as potential adsorbents for CO_2_ capture.^5–19^ POPs offer advantages over other porous materials, such as high chemical and thermal stability, low skeletal density, customizable architecture and pore size, and tunable functionality. In addition to the porous nature of POPs, the introduction of CO_2_-philic nitrogen functionalities, such as amine, imine, triazine, carbazole, azo, etc., into polymer structure has proven to be beneficial for enhancing CO_2_ capture capacity and selectivity.^20–28^ Among various nitrogen-rich moieties, the azo groups are especially attractive. As reported by Patel et al., azo-bridged POPs exhibit high chemical stability and excellent CO_2_/N_2_ selectivity with an increase in temperature, making them ideal candidates for postcombustion CO_2_ capture. Besides acting CO_2_-philic, it was suggested that azo groups reject N_2_ gas, rendering the framework “N_2_-phobic”.^23,24^

Due to their versatility as optoelectronic or catalytic moieties, along with rigid and tunable structure, porphyrins are also often incorporated into various porous materials.^29–33^ The basic pyrrole structure within the porphyrin can promote interactions with CO_2_, making porphyrin-based POPs effective CO_2_ adsorbents. The incorporation of both azo and porphyrin units into POPs resulted in azo-linked porphyrin-based porous polymer systems, which exhibit high CO_2_ uptake capacity (up to 3.98 mmol g^–1^ at 273 K and 1 bar) and excellent CO_2_/N_2_ selectivity (up to 64.3 at 273 K and 1 bar).^34–38^

Recently, we synthesized a series of azo-linked porphyrin-based porous organic polymers (APPs) featuring aromatic linkers of linear, bent and trigonal symmetry resulting in different topologies when connected to the tetraphenylporphyrin units. In addition, we studied systems in which tetraphenylporphyrin units were directly connected via azo bonds.^39^ APPs incorporating linkers showed significantly higher Brunauer–Emmett–Teller (BET) surface areas than those with directly connected tetraphenylporphyrin moieties. Although systems with good BET surface areas, especially those with flexible and nitrogen-rich linkers, also exhibited enhanced CO_2_ adsorption capacities, the differences in the CO_2_ uptakes of various APPs were much less pronounced compared to their BET surface areas. These indicated that favorable interactions of azo groups and pyrrole rings with CO_2_ molecules significantly improve the affinity of APPs for CO_2_. The experimental findings were corroborated with computational study, which provided additional insights into the influence of surface area and local landscape on the CO_2_ adsorption properties of APPs. Although based on ideal models with highly organized structures, the calculated CO_2_ adsorption capacities were comparable to experiments. The computational methodology used in our previous papers,^39–43^ combining periodic DFT optimizations and grand-canonical Monte Carlo (GCMC) simulations, together with the analysis of density plots and electrostatic potential maps, helped us to underscore the role of surface area and local environment on CO_2_ adsorption and suggested that porphyrin units act as extra CO_2_ binding sites when accessible on both sides of the plane.^39^

These results motivated us to examine whether the introduction of additional heteroatom-containing groups, besides those in porphyrin, could further enhance the CO_2_ adsorption performance of APPs. Following the presynthetic functionalization approach commonly used in MOF and POP chemistry, we employed linkers bearing heteroatom-containing groups to enable their direct incorporation into the APP framework. Considering the availability of functionalized linkers and the novelty of the resulting APPs, we focused on hydroxylated biphenyl (BP-OH) and carbonyl-bearing anthraquinone (AQ) to examine the influence of polar moieties. Furthermore, to probe the effect of steric hindrance near the azo linkages we also employed methylated biphenyl (BP-Me) and phenyl (Ph-Me) linkers. Therefore, we synthesized APP-BP-OH and APP-AQ (Figure 1) by heterocoupling of 5,10,15,20-tetrakis­(4-nitrophenyl)-21*H*,23*H*-porphyrin (TNPPR) and 4,4’-diaminobiphenyl-3,3′-diol (for APP-BP-OH) and 2,6-diaminoanthraquinone (for APP-AQ). Structural and functional characteristics of such functionalized APPs were compared to those of sterically hindered APP-BP-Me and APP-Ph-Me (Figure 1), which were synthesized by the heterocoupling of TNPPR and 3,3′-dimethylbiphenyl-4,4’-diamine (for APP-BP-Me) and 2,5-dimethylbenzene-1,4-diamine (for APP-Ph-Me). The obtained polymers were characterized in detail by IR and ^13^C CP/MAS NMR spectroscopy, powder X-ray diffraction (PXRD), elemental analysis, thermogravimetric analysis (TGA), scanning electron microscopy (SEM), nitrogen (N_2_) adsorption–desorption experiments, and computational methods.

**Figure 1:**
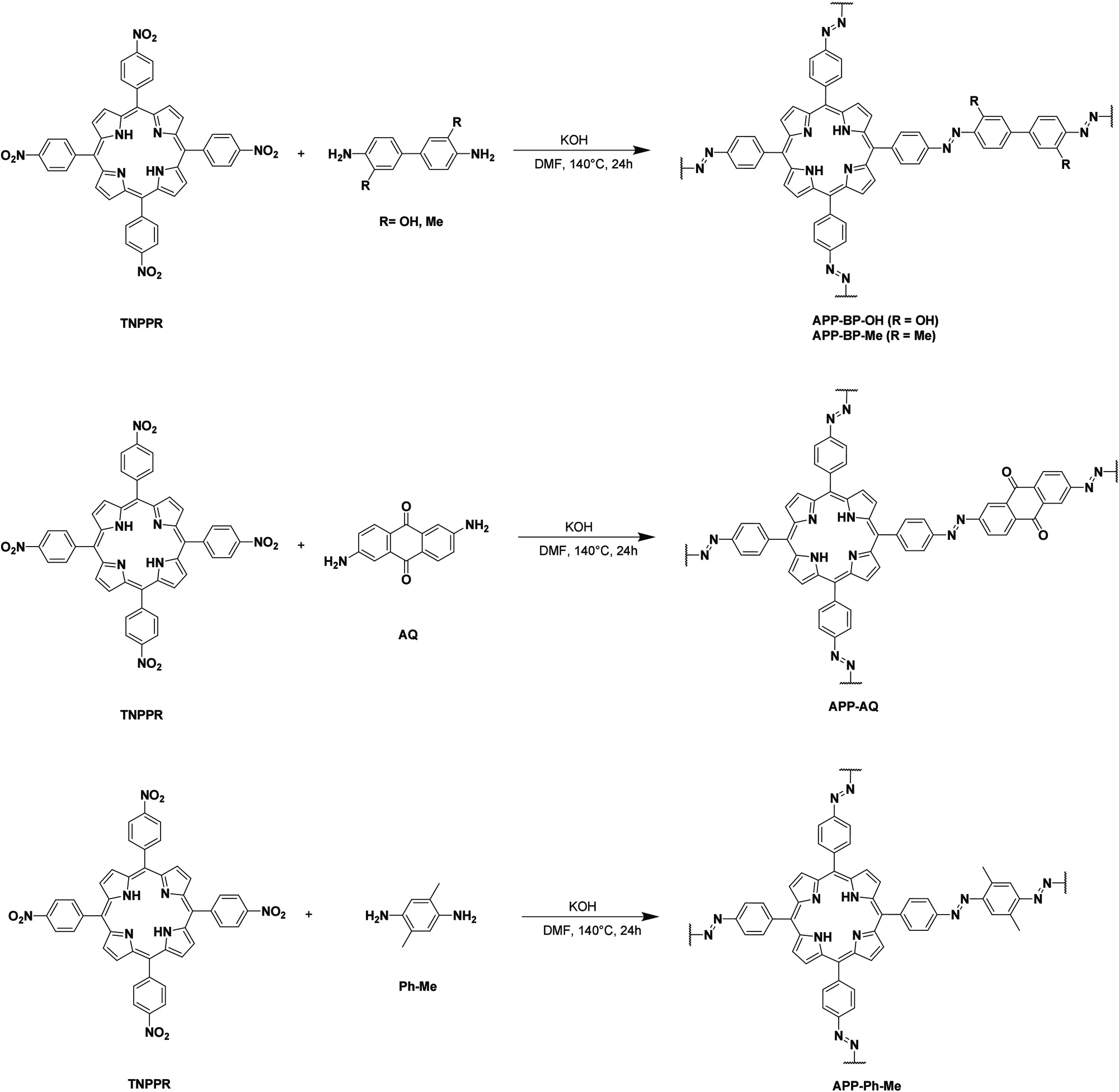
Synthesis of functionalized (APP-BP-OH and APP-AQ) and sterically hindered (APP-BP-Me and APP-Ph-Me) azo-linked porphyrin-based polymers (APPs) by heterocoupling of TNPPR and corresponding aromatic diamines.

## Results and Discussion

### Synthesis, Characterization, and CO_2_ Adsorption Performance of Polymers

Azo-bridged porphyrin-based polymers (APPs) bearing hydroxyl-functionalized biphenyl (APP-BP-OH), carbonyl-containing anthraquinone (APP-AQ), methylated biphenyl (APP-BP-Me) and methylated phenyl (APP-Ph-Me) moieties were synthesized by heterocoupling of 5,10,15,20-tetrakis­(4-nitrophenyl)-21*H*,23*H*-porphyrin (TNPPR) and corresponding aromatic diamino linkers (Figure 1 and Table S1). APPs were isolated in high yields (73–98%, Table S1) and were insoluble in common organic solvents. To confirm the formation of APPs and to study their properties, we have used a range of experimental methods. FTIR spectra of polymers were compared to spectra of starting TNPPR and diamino monomers (Figures 2, S3, S4 and S5). In the FTIR spectra of APPs, azo (N=N) stretching vibrations were observed around 1470 and 1400 cm^–1^. Their position is consistent with the N=N stretching modes in FTIR spectra of previously reported azo-bridged porphyrinic POPs.^36–39,44^ Furthermore, characteristic vibration bands of linkers were detected in the synthesized APPs. In the FTIR spectrum of APP-BP-OH, the C–O stretching vibration was identified at 1280 cm^–1^, while in the spectrum of APP-AQ characteristic carbonyl stretch appeared around 1680 cm^–1^. The asymmetric and symmetric stretching vibrations of unreacted nitro groups at 1520–1510 cm^–1^ and 1350–1340 cm^–1^, respectively, could be also observed in the spectra of the obtained polymers. In addition, FTIR spectra of all four APPs revealed bands around 3380 and 3300 cm^–1^ assigned to the N–H stretching vibrations of residual amino groups. This is consistent with the FTIR spectra of similar azo-bridged porphyrin-based POPs, which also showed bands attributed to unreacted nitro and amino groups.^34,36–39^

**Figure 2:**
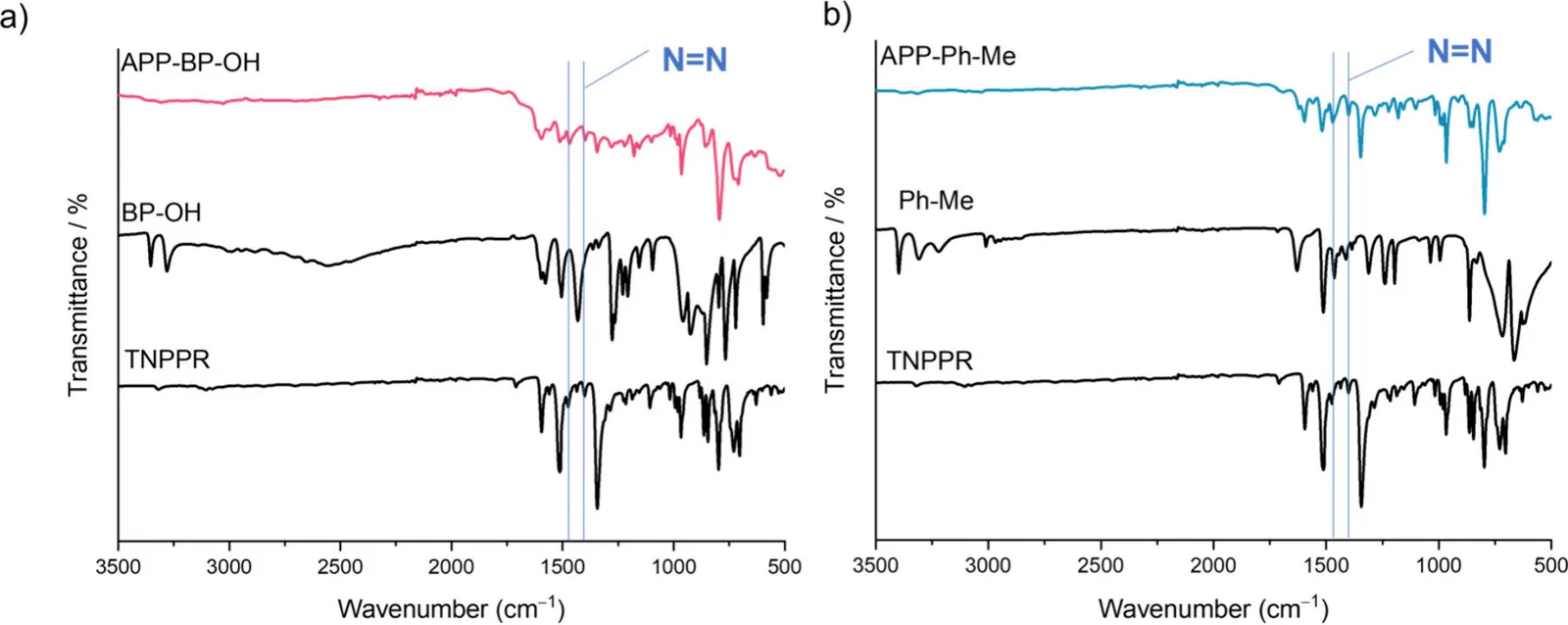
Comparison of FTIR spectra of (a) APP-BP-OH and starting nitro and amino monomers (TNPPR and BP-OH, respectively) and (b) APP-Ph-Me and starting nitro and amino monomers (TNPPR and Ph-Me, respectively). Vertical lines denote the characteristic azo (N=N) stretching vibrations, appearing near 1470 and 1400 cm^–1^.

Figure 3 shows ^13^C CP/MAS NMR spectra of APPs. Noteworthy, all the spectra showed signal around 150 ppm, which is assigned to the carbon directly attached to the azo group.^23,24,34,36,37,39,44,45^ Together with FTIR spectroscopy data, this confirmed the formation of azo bridges in the synthesized APPs. Furthermore, the ^13^C CP/MAS NMR spectra of polymers also presented aromatic carbon signals of building porphyrin and phenyl units, located around 145, 130, and 118 ppm. In the spectrum of APP-AQ, signal at 180 ppm appeared, characteristic of carbonyl carbon, while spectra of APP-BP-Me and APP-Ph-Me revealed signals of methyl carbon around 16 and 15 ppm, respectively.

**Figure 3:**
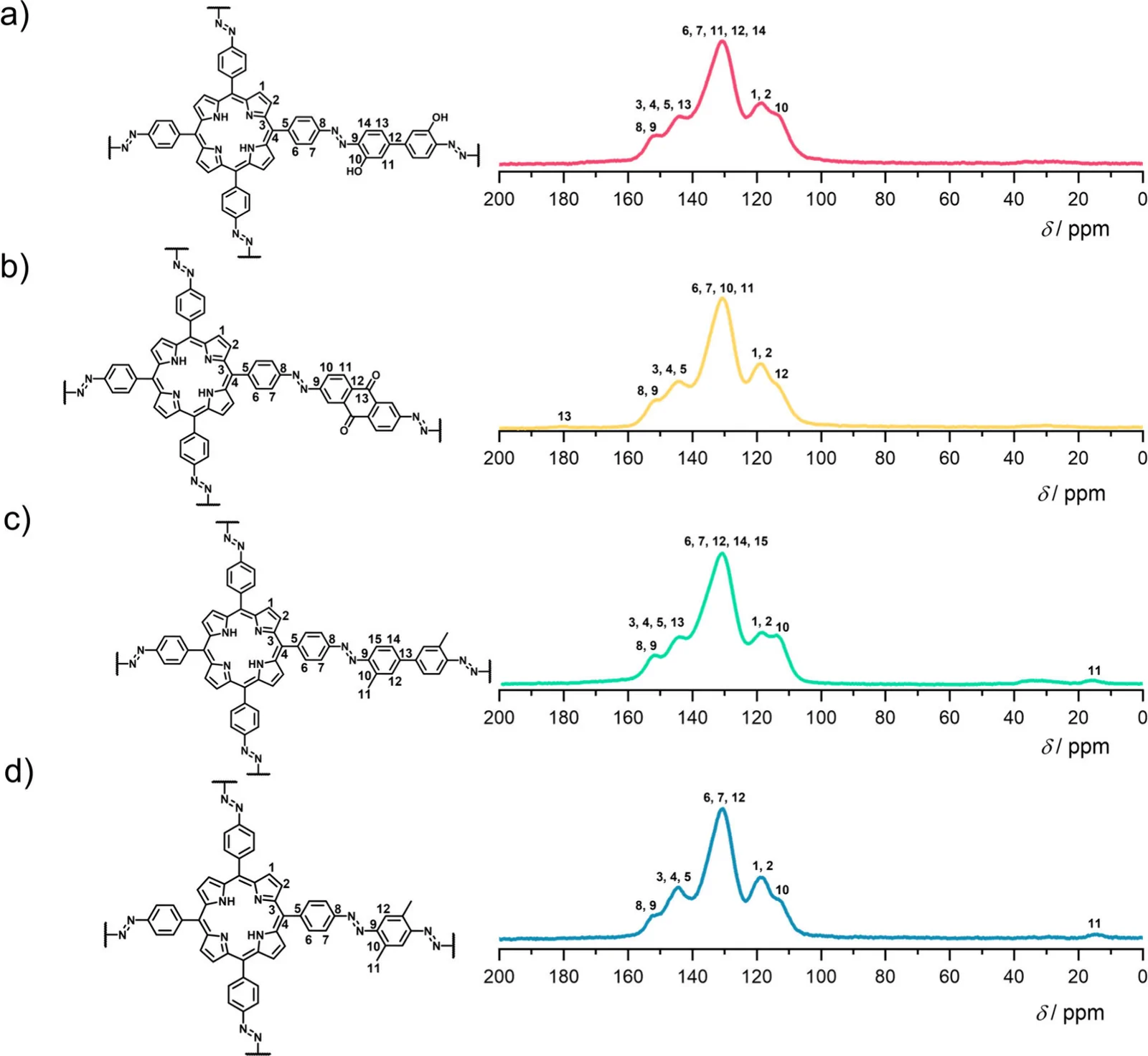
^13^C CP/MAS NMR spectra of (a) APP-BP-OH, (b) APP-AQ, (c) APP-BP-Me, and (d) APP-Ph-Me.

The broad diffraction peaks in the PXRD patterns of all four APPs indicated their amorphous nature (Figure 4). This is typical for azo-bridged POPs,^23,24,34–37,39,45–48^ where the irreversible formation of azo bonds prevents the formation of ordered structures.

**Figure 4:**
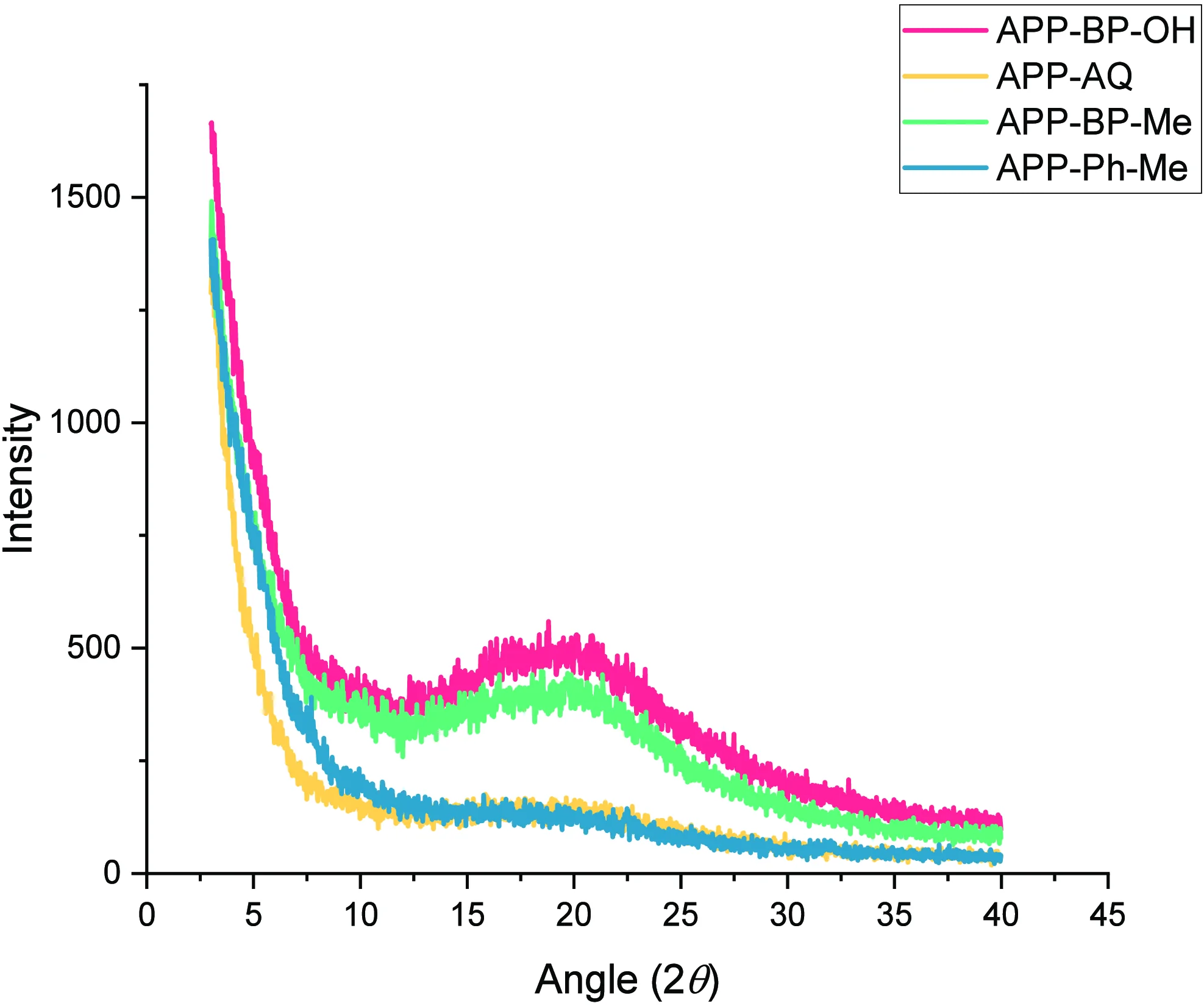
PXRD patterns of azo-linked porphyrin-based polymers APP-BP-OH, APP-AQ, APP-BP-Me, and APP-Ph-Me.

The thermal stability of APPs was determined by TGA. All samples were heated to 800 °C in N_2_ atmosphere at a heating rate of 10 °C min^–1^ and held under isothermal conditions at 800 °C for 15 min. TGA showed that all samples are stable to at least 200 °C and decompose in two main thermal events (Figures S6–S9). The first and smaller in magnitude (4–7%) begins at ∼ 200–240 °C depending on the sample. All samples show a second event with a greater decomposition rate starting at around 430 °C and reaching a maximum around 500 °C when the rate of decomposition decreases until ∼ 650 °C at which point the rate of decomposition stabilizes and a small decrease in mass is observed until the end of the isothermal step at 800 °C. The samples lose an additional 20–26% of their initial weight by 800 °C. APP-AQ and APP-Ph-Me exhibit similar thermal behavior, with initial decomposition starting around 240 °C and a major event peaking at ∼ 510 °C (Figures S7 and S9). APP-Ph-Me shows slightly higher weight loss (24.5%) than APP-AQ (20.2%). In contrast, APP-BP-OH and APP-BP-Me display lower thermal stability, with earlier onset of decomposition (∼210–230 °C) and greater weight loss, particularly for APP-BP-OH (Figures S6 and S8).

The observed elemental composition of APPs showed some deviations compared to the theoretical value. This is common to POPs and is probably caused by incomplete polymerization and moisture adsorption.^45,46,49^ As stated above, FTIR spectra of the obtained polymers revealed residual nitro and amino functional groups.

Figure 5 shows SEM images of APPs, revealing different morphologies characterized by agglomerated particles of various sizes.

**Figure 5:**
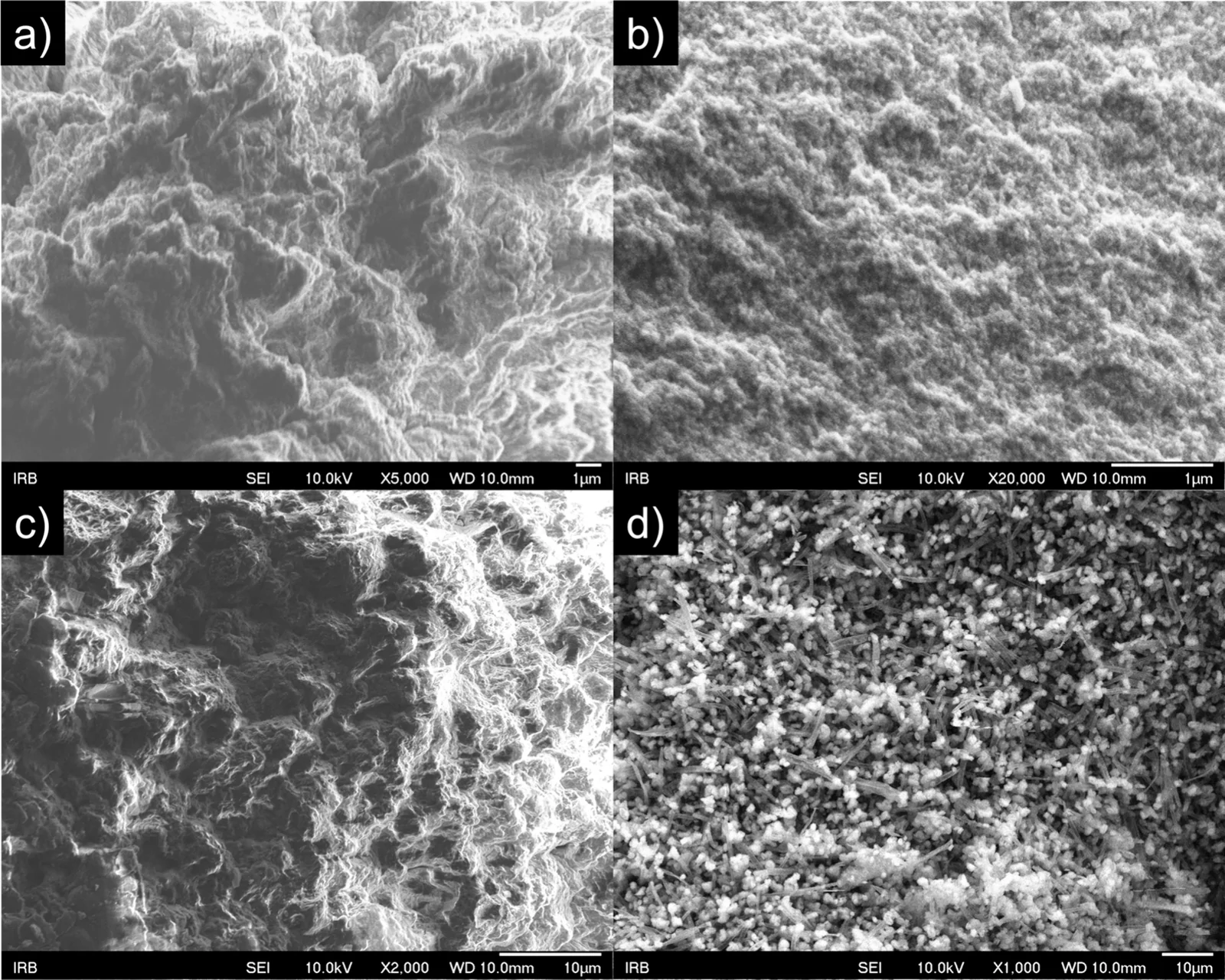
SEM images of (a) APP-BP-OH, (b) APP-AQ, (c) APP-BP-Me, and (d) APP-Ph-Me.

The porosity of APPs was investigated by N_2_ adsorption–desorption measurements at 77 K. All APPs exhibited type IV isotherms (Figure 6a),^50^ characteristic of mesoporous materials, with hysteresis loops related to capillary condensation and evaporation occurring within the mesopores. APP-BP-OH and APP-AQ showed moderate N_2_ uptake with a slightly reduced total pore volume (0.144 cm^3^ g^–1^ for APP-BP-OH and 0.145 cm^3^ g^–1^ for APP-AQ). APP-BP-Me displayed the lowest uptake and a small total pore volume (0.074 cm^3^ g^–1^). In contrast, APP-Ph-Me exhibited the highest uptake with a much larger pore volume (0.522 cm^3^ g^–1^). The pore size distribution further suggests the presence of micropores alongside mesopores, which is corroborated by a peak below 2 nm observed in all APP samples (Figure 6b). APP-Ph-Me clearly shows the highest mesopore contribution, followed by APP-BP-OH and APP-AQ showing moderate mesoporosity. In contrast, APP-BP-Me shows minimal mesopore content and a significant presence of larger pores extending beyond 50 nm. The specific surface areas were calculated using the BET model and were found to be 488, 410, 167, and 673 m^2^ g^–1^ for APP-BP-OH, APP-AQ, APP-BP-Me and APP-Ph-Me, respectively (Table 1). For comparison, the previously reported BET surface areas of similar azo-linked porphyrin-based POPs prepared by the same synthesis method, such as azo-CPP-1 to azo-CPP-7,^37^ and APP-1 to APP-6,^39^ are in the range from 197 to 591 m^2^ g^–1^ and 469 to 608 m^2^ g^–1^, respectively. The BET surface area of APP-Ph-Me (673 m^2^ g^–1^) is among the highest among the similar azo-linked porphyrin-based POPs synthesized by heterocoupling of TNPPR and aromatic diamines.^34,36–38^ The incorporation of hydroxyl (APP-BP-OH) and especially methyl (APP-BP-Me) groups into the biphenyl linker resulted in reduced porosity compared to the previously reported Azo-CPP-3 (514 m^2^ g^–1^)^37^ with an unsubstituted biphenyl unit.

**Figure 6:**
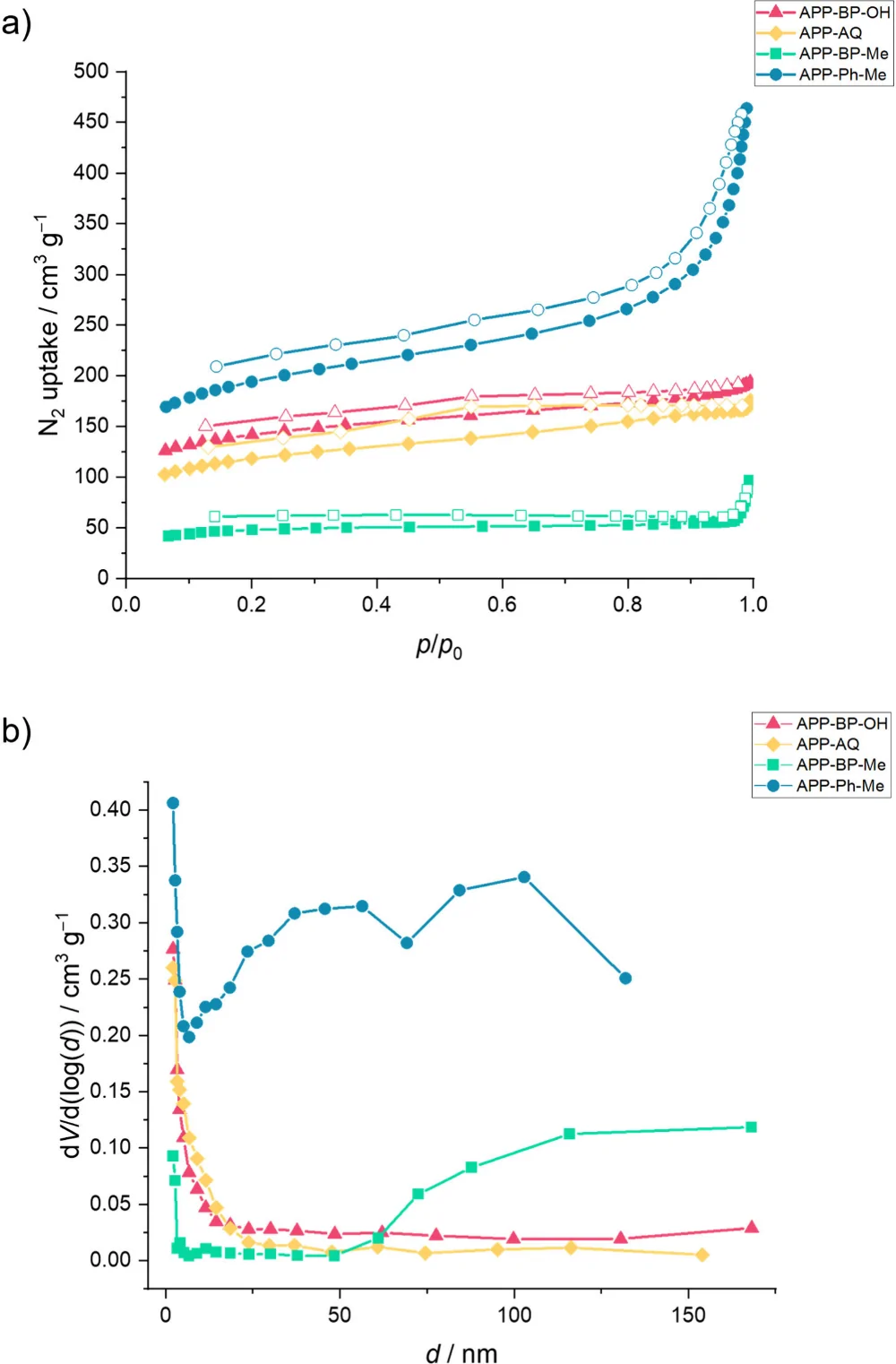
(a) N_2_ adsorption–desorption isotherms measured at 77 K and (b) pore size distributions of APP-BP-OH, APP-AQ, APP-BP-Me, and APP-Ph-Me. The adsorption and desorption isotherms in panel a are depicted with filled and empty markers, respectively.

**Table 1:** Comparison of BET Surface Areas and CO_2_ Uptakes for Synthesized APPs and Other State-of-the-Art Azo and/or Porphyrin-Containing POPs

compound	BET surface area (m^2^ g^–1^)	CO_2_ uptake^a^ (mg g^–1^)
APP-BP-OH	488	49
APP-AQ	410	40
APP-BP-Me	167	41
APP-Ph-Me	673	41
APP-1 ^39^	598	41
Azo-CPP-2 ^37^	439	55.4
Azo-2 ^34^	675	89.7^b^
Azo-PPor-1 ^36^	750	108^c^
NAP1 ^35^	1095	99.3^b^
Por-Py-CMP^54^	1014	82^b^
Azo-Por-Bpy-POP^51^	561	39.1^b^
PNOP-1 ^52^	830	63.1^b^
ALP-1 ^45^	1235	143^b^
Azo-COP-2 ^24^	729.6	67.3^b^
Azo-Py^55^	1259	127^b^
Azo-POF-1 ^53^	712	82.9^b^

a At 303 K.

b At 298 K.

c At 273 K.

The CO_2_ adsorption performance of herein synthesized APPs was investigated by TGA at approximately 30 °C (303 K) (Figures 7, S10 and S11). APP-Ph-Me and APP-AQ show similar adsorption profiles with relatively sharp changes in weight during the transition between N_2_ and CO_2_ cycles (Figures 7b and S10). They reach maximum values during CO_2_ cycles and transition back to baseline values during N_2_ cycles in less than 5 min. APP-BP-OH and APP-BP-Me have slightly different adsorption profiles (Figures 7a and S11). Both samples show a sharp increase in weight during CO_2_ cycles to about 90% of the maximum value followed by a gradual increase to maximum values with APP-BP-Me reaching a plateau after around 15 min and APP-BP-OH showing a slow increase until the end of the first CO_2_ cycle. Both samples show a more pronounced and sharper increase in weight during the second CO_2_ cycle. When switching to N_2_ cycles both samples show a rapid decrease in weight, which stops before reaching baseline values. APP-BP-Me retains an increased weight corresponding to ∼100.2% of its initial weight during the N_2_ cycles followed by a very gradual decline until the end of the cycle. APP-BP-OH retains an increased weight corresponding to ∼100.4% of its initial weight during the N_2_ cycles and shows a negligible decrease in weight until the end of the cycle.

**Figure 7:**
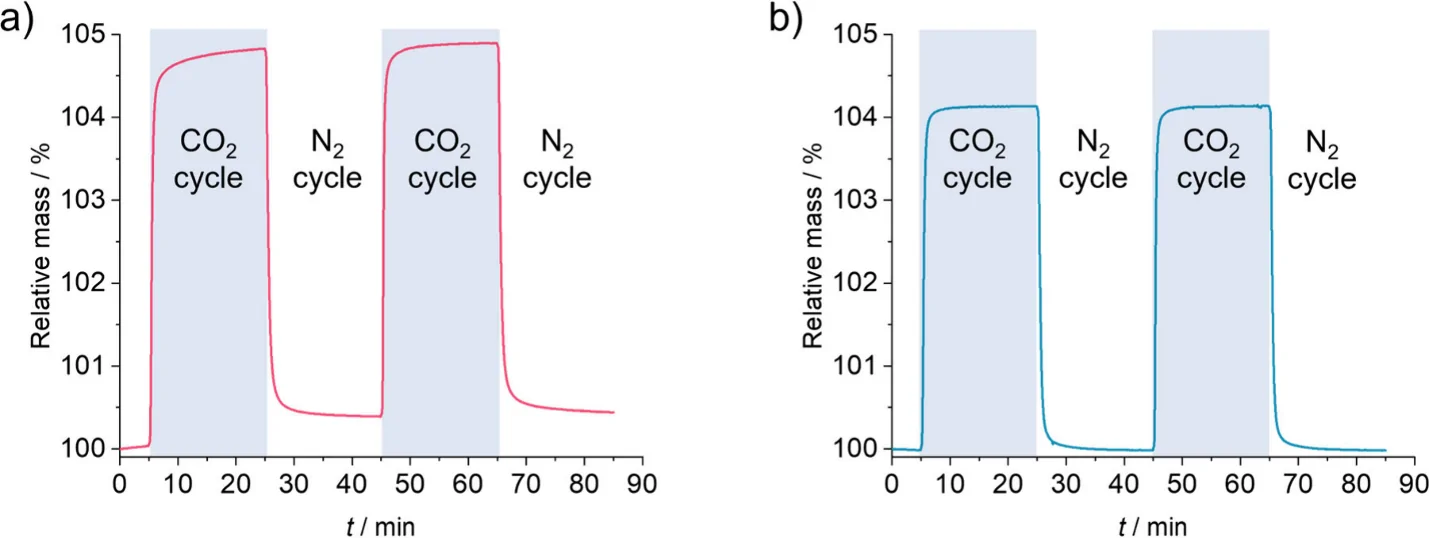
Representative thermogravimetric CO_2_ adsorption and desorption profiles of (a) APP-BP-OH and (b) APP-Ph-Me at approximately 30 °C (303 K).

The highest CO_2_ adsorption capacity of 49 mg g^–1^ was observed for APP-BP-OH, which combines a biphenyl linker with polar hydroxyl groups (Table 1). For comparison, the CO_2_ uptake of similar azo-linked porphyrin POP, Azo-CPP-3, with unfunctionalized biphenyl linker, is 39.9 mg g^–1^ at 303 K.^37^ The other APPs displayed comparable CO_2_ uptake values of 40 mg g^–1^ for APP-AQ, and 41 mg g^–1^ for APP-BP-Me and APP-Ph-Me. In general, the differences in CO_2_ uptakes (from 40 to 49 mg g^–1^) of these APPs are much less pronounced than their BET surface areas (from 167 to 673 m^2^ g^–1^), indicating that favorable interactions between the CO_2_ molecules and nitrogen-rich azo-linked porphyrin-based polymer frameworks have the most significant impact on the affinity of these systems for CO_2_. In addition to the contribution of nitrogen-rich porphyrin and azo moieties, the incorporation of polar hydroxyl groups can introduce additional favorable interactions between CO_2_ and framework. Finally, the nature of the linker itself also contributes to CO_2_ adsorption. A direct comparison between APP-BP-Me and APP-Ph-Me shows that APP-BP-Me, despite its significantly lower surface area (167 m^2^ g^–1^), exhibits a CO_2_ uptake (41 mg g^–1^) comparable to that of APP-Ph-Me and APP-AQ, suggesting that the biphenyl linker plays a role in enhancing pore accessibility and local adsorption environment. Functionalized and sterically hindered APPs have the slightly enhanced (APP-BP-OH) or comparable (APP-AQ, APP-BP-Me and APP-Ph-Me) CO_2_ uptakes capacities to previously reported APP-1 and APP-2 containing flexible methylene and ethylene bridge (41 mg g^–1^ and 38 mg g^–1^, respectively, at 306 K), and APP-5 with melamine linker (38 mg g^–1^ at 306 K), while their affinity for CO_2_ is higher than that of e.g., APP-3 with sulfur (29 mg g^–1^ at 306 K) and APP-4 with carbonyl (30 mg g^–1^ at 306 K) bridge.^39^ Furthermore, the CO_2_ adsorption capacities of herein studied APPs are comparable to e.g., Azo-CPPs (39.9–55.4 mg g^–1^ at 303 K),^37^ Azo-Por-Bpy-POP (39.1 mg g^–1^ at 298 K)^51^ and Azo-Por-Dadp-POP (36.8 mg g^–1^ at 298 K),^51^ representing azo-linked porphyrin-based POPs incorporating linkers between tetraphenylporphyrin units; porphyrin-containing POPs such as PNOP-2 (55.2 mg g^–1^ at 298 K);^52^ and azo-linked POPs such as Azo-COP-3 to azo-COP-11 (49–58 mg g^–1^ at 298 K),^23^ and Azo-POF-2 (55.1 mg g^–1^ at 298 K).^53^ Furthermore, they are higher compared to APPs with tetraphenylporphyrin units directly connected via azo bonds (15–28 mg g^–1^ at 306 K).^39^ However, as shown in Table 1, they are lower when compared to some other best-performing azo-linked porphyrin-based POPs, such as Azo-1 to Azo-3 (66.0–89.7 mg g^–1^ at 298 K),^34^ Azo-PPor-1 to Azo-PPor-3 (95–108 mg g^–1^ at 273 K),^36^ and NAP1 and NAP2 (99.3 and 84.6 mg g^–1^, respectively, at 298 K);^35^ POPs containing porphyrin moieties, such as Por-Py-CMP (82 mg g^–1^ at 298 K),^54^ and PNOP-1 (63.1 mg g^–1^ at 298 K);^52^ and azo-linked POPs, such as ALPs (68–143 mg g^–1^ at 298 K),^45,46^ Azo-COP-1 and Azo-COP-2 (65.3 and 67.3 mg g^–1^, respectively, at 298 K),^24^ Azo-Py (127 mg g^–1^ at 298 K),^55^ and Azo-POF-1 (82.9 mg g^–1^ at 298 K).^53^

### Computational Study

Recently, we highlighted the potential of 2D-layered APPs for CO_2_ binding, particularly when porphyrin rings are accessible and favorably oriented for interactions with CO_2_ molecules.^39^ The computational study suggests that this can be achieved by shifting the neighboring layers from a perfectly stacked AA (eclipsed) to an AB (staggered) configuration. However, configurations with staggered neighboring layers are less favorable than their eclipsed counterparts. Porphyrin rings directly linked via azo bonds typically lead to the formation of rhombohedral pores.^39^ The insertion of linear linkers connecting the tetragonal porphyrin units primarily increases pore size without altering the overall pore shape within a single 2D layer. In contrast, bent linkers such as –Ph–CH_2_–Ph– or –Ph–CO–Ph– induce the formation of distorted, rectangular-like pores.^39^

To gain a comprehensive understanding of the structural and CO_2_ adsorption properties of the newly synthesized APPs, we carried out an extensive computational study. Although the synthesized materials were amorphous, we assumed highly ordered AA (eclipsed) configurations for the purposes of computational analysis, consistent with the approach adopted in our previous studies.^39–41^ This study focuses on how various functionalized and sterically hindered linkers – particularly those containing hydroxyl, carbonyl and methyl groups – affect the structural organization and CO_2_ adsorption performance of APPs. Figure 8a–8d shows the optimized geometries of the four investigated systems, all exhibiting typical rhombohedral pores. While the calculated CO_2_ uptake values (Table 2 and Figure 8e) are notably lower than those observed experimentally (Table 1), the trends are consistent. For instance, APP-BP-OH, which incorporates polar hydroxyl groups, demonstrates slightly enhanced CO_2_ uptake, both computationally (25 mg g^–1^) and experimentally (49 mg g^–1^). The calculated CO_2_ uptake values are further corroborated by the calculated zero-loading enthalpies of adsorption (from –14 to –15 kJ mol^–1^, Table 2), indicating physisorption-dominated adsorption, with CO_2_ stabilized by weak noncovalent interactions.

**Figure 8:**
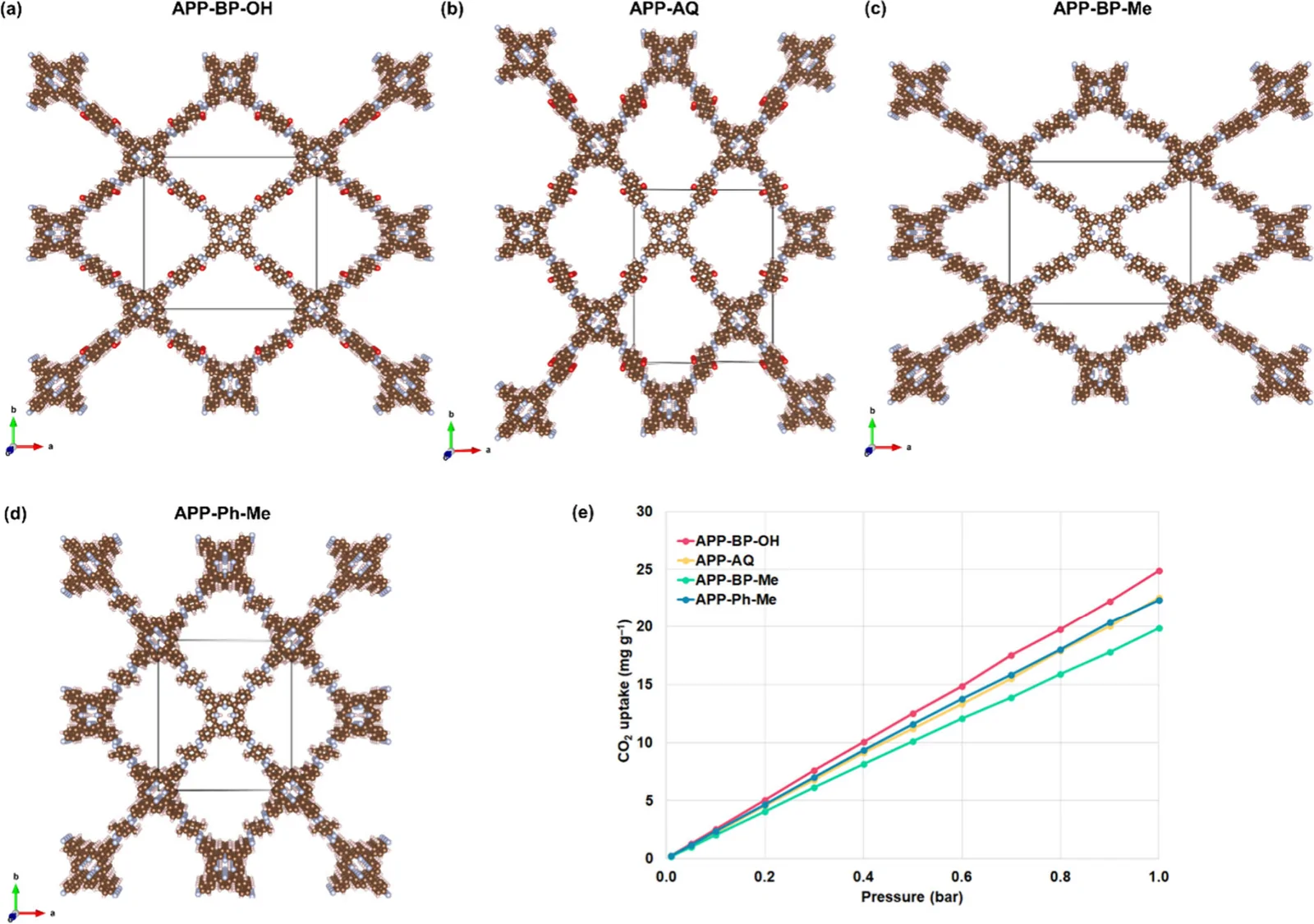
Optimized geometries (PBE-D3/pob-TZVP-rev2) of 2D-layered (a) APP-BP-OH, (b) APP-AQ, (c) APP-BP-Me, and (d) APP-Ph-Me. (e) CO_2_ adsorption isotherms of modeled APPs at 298 K.

**Table 2:** Calculated Average Surface Areas, CO_2_ Uptakes (at 298 K and 1 bar), and CO_2_ Enthalpies of Adsorption (at 298 K) of the APPs

compound	average surface area (m^2^ g^–1^)	CO_2_ uptake (mg g^–1^)	enthalpy of adsorption (kJ mol^–1^)
APP-BP-OH	1750	25	–14.9
APP-AQ	1699	23	–14.1
APP-BP-Me	1805	20	–13.6
APP-Ph-Me	1660	23	–14.8

The primary reason for the discrepancy between calculated and experimentally determined surface areas lies in the assumption of perfectly ordered geometries, which was made to simplify the computational study. Nevertheless, in both cases, there is no clear correlation indicating that an increase in surface area necessarily leads to enhanced CO_2_ uptake within the framework. For example, among the investigated APPs, the methylated derivative APP-Ph-Me exhibits the highest experimentally determined surface area (673 m^2^ g^–1^), while APP-BP-Me shows the highest surface area determined computationally (1805 m^2^ g^–1^). However, their CO_2_ uptake values of 41 mg g^–1^ (experimentally obtained for APP-Ph-Me) and 20 mg g^–1^ (calculated for APP-BP-Me) are not the highest in the series. This suggests that CO_2_ adsorption performance of APPs is influenced not only by surface area but also by the local chemical environment created by functional groups introduced on the linkers. To test this hypothesis, we have calculated electrostatic potential (ESP) maps for the four APPs and identified regions with the most negative ESP values (Figures 9a and S13), which serve as potential binding sites for CO_2_ molecules. In methylated derivatives, APP-BP-Me and APP-Ph-Me, the nitrogen atoms of the pyrrole rings, along with those in the azo linkages, exhibit the most negative ESP values. Due to the AA (eclipsed) configuration, the regions above and below the porphyrin rings are sterically inaccessible for interactions with CO_2_. The introduction of additional heteroatom functionalities, such as hydroxyl or carbonyl groups, can significantly alter the electron distribution and create new accessible binding sites, thereby enhancing the CO_2_ adsorption capacity of the framework. The ESP value on hydroxyl oxygen in APP-BP-OH reaches –134 kJ mol^–1^ e^–1^, while the carbonyl oxygen in APP-AQ exhibit even more negative potential of –166 kJ mol^–1^ e^–1^, highlighting these sites as particularly favorable for CO_2_ binding. Furthermore, the introduction of hydroxyl and carbonyl groups in APP-BP-OH and APP-AQ, respectively, withdraws electrons from the porphyrin core and azo linkages, resulting in less negative ESP values on their nitrogen atoms when compared to the effect of methyl groups in APP-BP-Me and APP-Ph-Me. This trend is further supported by the density plots showing the spatial distribution of CO_2_ molecules (Figures 9b and S16). While the methylated APPs exhibit high adsorption density primarily around the azo linkages and accessible regions of porphyrin rings, APP-BP-OH and APP-AQ also display CO_2_ distribution near the hydroxyl and carbonyl groups. The effect is particularly evident for the hydroxyl group, as highlighted by the cyan colored regions in the density map. Additional confirmation comes from the optimization of a series of CO_2_···APP configurations involving hydroxyl, carbonyl and methyl groups (Figures 9c and S21–S23). In each series, the most stable conformers correspond to those in which CO_2_ molecule interacts directly with heteroatoms. Notably, the interaction with the hydroxyl group is stronger (−31 kJ mol^–1^) than with carbonyl group (−27 kJ mol^–1^). These findings are in good agreement with the CO_2_ uptake values calculated using the GCMC method – slightly stronger interactions correspond to slightly higher CO_2_ uptake values in APP-BP-OH (25 mg g^–1^), while weaker interactions in APP-AQ are reflected in a slightly lower CO_2_ uptake (23 mg g^–1^).

**Figure 9:**
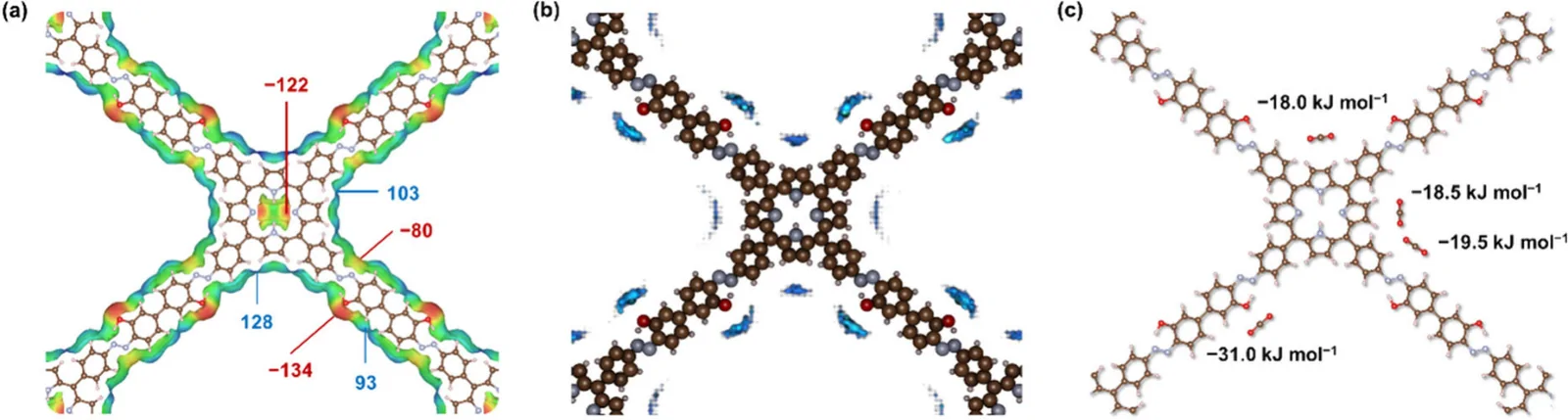
Computational analysis of potential CO_2_ binding sites in APP-BP-OH. (a) Electrostatic potential map highlighting areas of negative (red) and positive (blue) potential, calculated in kJ mol^–1^ e^–1^ and visualized on the 0.002 au electron density isosurface. (b) Density plot illustrating the average distribution of adsorbed CO_2_ molecules, calculated at 298 K and 1 bar. (c) Interaction energies (in kJ mol^–1^) for selected CO_2_···APP-BP-OH complexes, representing the interaction between a single CO_2_ molecule and the framework.

Since highly ordered structures of APPs have not been confirmed experimentally and are instead found to be amorphous, we investigated how shifting neighboring layers affects CO_2_ adsorption. Due to the lack of long-range order in amorphous structures, a greater proportion of porphyrin rings are expected to be sterically unhindered. To explore this effect, three configurations were examined, AA (eclipsed) and AB (staggered) as limiting cases – representing no shift and maximal shift between neighboring layers, respectively – and AA’ (serrated) configuration, optimized from a geometry with a 50% interlayer offset (Tables S5 and S6, Figure S17). The AA configuration, characterized by the largest but uniform pores, exhibits the lowest surface area (1750 m^2^ g^–1^) and CO_2_ uptake (25 mg g^–1^). In contrast, the AA’ configuration, which shows smaller pores, achieves the highest surface area (2716 m^2^ g^–1^) and CO_2_ uptake (213 mg g^–1^). The AB configuration shows bimodal pore size distribution, but reduced surface area (1894 m^2^ g^–1^) and intermediate CO_2_ uptake (85 mg g^–1^). Density plots of CO_2_ distribution further clarify the observed trends (Figure S18). Shifting from AA to AA′ increases accessible surface area and exposes both faces of the porphyrin rings, while preserving access to hydroxyl groups and finally enhancing the CO_2_ adsorption. Further shifting to AB introduces additional pores but reduces accessibility, making porphyrin rings the dominant binding sites, even surpassing hydroxyl groups in interaction strength. Although AA′ and AB are energetically less favorable, and thus not expected to be formed, these idealized models underscore the importance of pore accessibility, size distribution, and local chemical environment in determining CO_2_ adsorption performance.

## Experimental Section

### Materials and Methods

All chemicals were used as is. All aromatic amines were purchased from the suppliers. TNPPR was characterized by solution-state ^1^H NMR spectroscopy. Spectra were recorded on a Bruker Ascend 400 MHz NMR spectrometer at 25 °C. DMSO-d6 was used as a solvent and TMS as an internal standard for chemical shifts. The synthesized polymers were characterized by solid-state ^13^C CP/MAS NMR spectroscopy, IR spectroscopy, PXRD and elemental analysis. Solid-state ^13^C CP/MAS NMR spectra were recorded on a Bruker Avance Neo 400 MHz NMR spectrometer and Bruker Avance III HD 400 MHz NMR spectrometer at spinning rates of 10 and 15 kHz, respectively. FTIR spectra were recorded on a PerkinElmer UATR two spectrometer in the spectral range between 4000 cm^–1^ and 400 cm^–1^ at a resolution of 4 cm^–1^, averaging 10 scans per spectrum. Elemental analysis was provided by the Analytical Services Laboratory of the Rud̵er Bošković Institute, Zagreb, Croatia. PXRD diffractograms were recorded on a Malvern Panalytical Aeris powder diffractometer in Bragg–Brentano geometry with PIXcel1D detector. The presence of azo groups in APPs was confirmed by FTIR and ^13^C CP/MAS NMR spectroscopy. Thermogravimetric analysis was carried out using a simultaneous TGA-DTA analyzer Mettler-Toledo TGA/DSC 3+. Samples were placed in alumina pans (70 μL) and heated in flowing nitrogen (50 mL min^–1^) from 30 °C up to 180 °C at a rate of 10 °C min^–1^ and held in isothermal conditions for 10 min at 180 °C to remove traces of solvents. Afterward, samples were cooled to room temperature and heated in flowing nitrogen (50 mL min^–1^) from 25 °C up to 800 °C at a rate of 10 °C min^–1^ and held in isothermal conditions for 15 min at 800 °C. CO_2_ sorption experiments were carried out by following a previously reported procedure with minor modifications in experimental conditions.^56^ Before performing CO_2_ adsorption experiments, a 70 μL alumina pan was filled with a fresh sample, heated to 100 °C at a heating rate of 20 °C min^–1^ in nitrogen atmosphere (flow rate 150 mL min^–1^) and held at 100 °C for 30 min to dry the sample. After drying, CO_2_ adsorption was measured by switching between N_2_ atmosphere and CO_2_ atmosphere in 20 min intervals (flow rates for both gases were 150 mL min^–1^) at ∼30 °C. The measured sample temperature varied around 30 °C during CO_2_ cycles. To correct for different buoyancy effects on the TG scale and alumina pan, a baseline curve was recorded under the same experimental conditions using an empty alumina pan and subtracted from the measured curve. Data collection and analysis were performed using the program package STARe Software 16.40 MettlerToledo GmbH. SEM images were taken using a thermal field-emission scanning electron microscope (FE-SEM, model JSM-7000F, JEOL Ltd., Tokyo, Japan) operating at an accelerating voltage of 10 kV and a working distance of 10 mm. The specific surface area was determined from N_2_ gas adsorption–desorption data obtained with Micromeritics ASAP-2000 at 77 K. Before analysis, samples were degassed at 150 °C under a dynamic vacuum of 7 mPa. The adsorption data were used to calculate the surface area with the BET model, using data points within the linear region of the BET isotherm. The pore size distribution was determined using the Barrett–Joyner–Halenda (BJH) method.

### Synthesis

The details of the synthetic procedures and products data are provided in the SI. 5,10,15,20-tetrakis­(4-nitrophenyl)-21*H*,23*H*-porphyrin (TNPPR), was synthesized by the modified procedures described in the literature.^57^ APP-BP-OH, APP-AQ, APP-BP-Me and APP-Ph-Me were synthesized via heterocoupling synthetic methods by adopting the procedures reported in the literature.^39^

### Computational Study

Computational study started by generating the APPs initial geometries by modifying the most stable conformation of previously reported azo-linked porphyrin-based polymer APP-7.^39^ For APP-BP–OH, two additional stacking configurations (inclined and serrated) were produced to analyze the impact of stacking configuration on CO_2_ adsorption isotherms. Additionally, several CO_2_···APPs complexes were generated by inserting a single CO_2_ molecule inside a 3D crystal structure at potential binding sites. The geometries of these compounds were generated as 3D crystal structures, with their space group symmetry determined and confirmed using PLATON.^58^ All structures are visualized using the VESTA program.^59^

All periodic density functional theory (DFT) calculations were performed in CRYSTAL23 ^60^ using triple-ζ basis set pob-TZVP-rev2 ^61^ and the PBE functional^62^ with Grimme’s D3 correction to better describe weak dispersive interactions.^63^ Input files for CRYSTAL23 were created from cif files using the cif2cell package.^64^ A full optimization of unit cell parameters and atom coordinates was performed, with default convergence criteria applied. The total energy convergence threshold was set to 10^–7^ and the truncation parameters for computing Coulomb and exchange integrals were increased to (8 8 8 8 16) for SCF calculations. The SHRINK parameter, which determinates the size of reciprocal space in the Pack-Monkhorst scheme, was set to 2 × 2 × 8. For all DFT-optimized structures, lattice parameters, atomic coordinates and relevant structure data are provided in the Supporting Information. The interaction energies of CO_2_ ···APPs were calculated using the formula Δ*E* = *E*_*CO*_2_···*APP*_-(*E*_*CO*_2__+*E*_*APP*_). The values were corrected for basis set superposition error (BSSE) using the counterpoise correction method proposed by Boys and Bernardi.^65^ To generate electronic density and electrostatic potential (ESP) values, we used two hundred points along the first lattice axis and equally spaced for other periodic dimensions. The required cube files with ESP values were calculated using CRYSTAL23. The obtained ESP values were plotted on the 0.002 au isodensity surface and the minimal and maximal values for the unit cells were compared.

The accessible surface area, framework density, available pore volume and pore size distribution were computed using the Monte Carlo sampling method with a spherical probe of 1.55 Å (corresponding to the van der Waals radius of nitrogen), employing the Zeo++ software.^66^ Utilizing ESP values calculated by CRYSTAL23, the net atomic charges were determined using the REPEAT method.^67^ GCMC simulations were performed using the RASPA code^68^ to obtain CO_2_ adsorption isotherms at 298 K and pressure ranging from 0 to 1 bar. The selectivity of APPs was calculated for binary mixture adsorption isotherms CO_2_/N_2_ molar ratio of 15: 85 at 298 K. Interactions between CO_2_ or N_2_ molecules and the framework were modeled using a combination of site–site Lennard-Jones (LJ) potential and Coulombic interactions. Lennard-Jones interactions between different atoms were described using Lorentz-Berthlot mixing rules. Default cutoff values provided by the RASPA code were applied for both LJ interactions and the short-range component of Coulombic interactions, while the long-range were assessed by the Ewald summation method with a default relative precision of 10^–6^. Gas molecules were represented as three-site models based on the TraPPE force field,^69^ while other atoms were modeled using the DREIDING force field.^70^ The pressure was converted to fugacity using the Peng-Robison equation of state to calculate the chemical potential. All simulations were performed on 2 × 2 × 8 unit cells, ensuring that all perpendicular cell lengths exceeded twice the default cut-of distance of 12 Å, as required for valid GCMC simulation cells. Four different MC moves (translation, rotation, reinsertion and swap) were allowed for gas molecules, while framework atoms were kept fixed. For equilibration and production phases, more than 10^6^ cycles were employed. The enthalpies of adsorption were determined following the RASPA manual, by sampling a single adsorbate molecule in the rigid framework, corresponding to the zero-loading (infinite-dilution) limit. Density pictures of CO_2_ adsorption at 298 K and 1 bar were obtained as described in the RASPA manual and visualized in the iRASPA advanced visualization package.^71^ More details on framework adsorption properties can be found in Supporting Information.

## Conclusion

A series of azo-bridged porphyrin-based polymers (APPs) with functionalized (APP-BP-OH and APP-AQ) and sterically hindered (APP-BP-Me and APP-Ph-Me) aromatic linkers were synthesized and characterized. FTIR, NMR, and PXRD confirmed the presence of azo linkages and the amorphous nature of the synthesized APPs. Thermal analysis showed that all materials remain stable up to about 200 °C. Surface area and gas sorption measurements revealed that the functionalization of the linkers has an important influence on porosity and CO_2_ adsorption properties. APP-Ph-Me displayed the highest surface area (673 m^2^ g^–1^) and well-developed mesoporosity. Although APP-Ph-Me exhibits the highest BET surface area, the differences in CO_2_ uptake among the investigated APPs are much less pronounced, indicating that CO_2_ adsorption is governed by a combination of different factors, like pore accessibility, linker topology and local chemical environment besides the surface area alone. Introduction of hydroxyl groups in APP-BP-OH lowered the surface area (488 m^2^ g^–1^) but led to an increased CO_2_ uptake (49 mg g^–1^ compared to 41 mg g^–1^ for APP-Ph-Me). This suggests that CO_2_ adsorption is also influenced by the contribution of polar hydroxyl groups and biphenyl linkers, which provide additional adsorption sites alongside nitrogen rich moieties. Computational studies confirmed that heteroatom-containing linkers, such as hydroxyl groups in APP-BP-OH and carbonyl groups in APP-AQ, generate favorable CO_2_ adsorption sites. In contrast, methylated systems (APP-BP-Me and APP-Ph-Me) depend mainly on less accessible porphyrin and azo regions. Additionally, stacking models suggested that changes in interlayer arrangement can further influence the accessibility of porphyrin rings and functional groups, helping to explain the behavior observed in the amorphous polymers.

In summary, these findings underline the effects of pore accessibility and functionalization of building units in determining the adsorption properties of the investigated APPs. The ability to tune the porosity and the local binding environment offers useful guidelines for creating advanced porous materials suitable for selective CO_2_ capture and related applications.

## Supplementary Material



## Notes

Detailed experimental procedures, ^1^H NMR and FTIR spectra, TGA results, and results of DFT and GCMC studies (PDF)
