# Supplementary material for: Tuning Linkers in Azo-Linked Porphyrin-Based Porous Organic Polymers for Enhanced CO2 Capture
Source: Langmuir. 2026 Feb 1;42(6):4641–52. doi: 10.1021/acs.langmuir.5c05572 (PMC13739203; doi:10.1021/acs.langmuir.5c05572)
Supplement: Supplementary file 1 [file la5c05572_si_001.pdf]

Tuning linkers in azo-linked porphyrin-based porous organic polymers for enhanced CO_2 capture

Matija Popović, Tea Frey, Mladen Borovina, Ivan Kodrin, and Ivana Biljan**

University of Zagreb Faculty of Science, Department of Chemistry, Horvatovac 102a, HR-10000 Zagreb, Croatia

Supplementary Materials

Contents

1. Experimental 2
- **1.1. Synthesis of TNPPR** 2
- **1.2. Synthesis and characterization of APPs** 4
- **1.3. Thermogravimetric analysis** 7

2. Computational studies of APPs model systems 11
- **2.1. Optimized geometries of APPs** 11
- **2.2. Electrostatic potential maps** 12
- **2.3. Calculated adsorption isotherms and density plots** 13
- **2.4. Stacking modes of APP-BP-OH** 15
- **2.5. Interaction energies calculated for CO_2···APPs complexes** 18
- **2.6. Fractional atomic coordinates and unit cell parameters** 20

References 25

1. Experimental

1.1. Synthesis of TNPPR

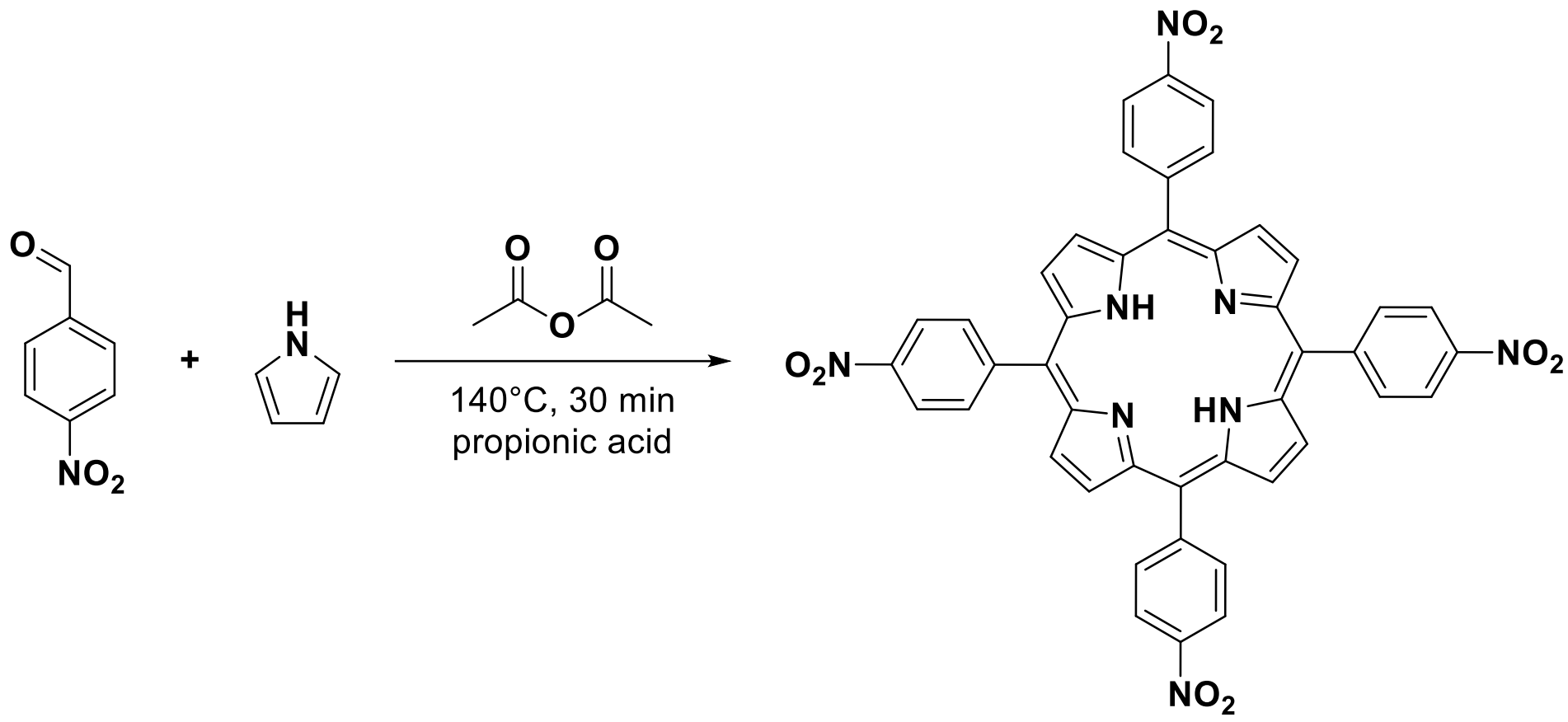

Figure S1. Synthesis scheme of TNPPR.

Synthesis of 5,10,15,20-tetrakis(4-nitrophenyl)-21*H*,23*H*-porphyrin (TNPPR) was carried out according to a procedure previously described in the literature.[1] p-Nitrobenzaldehyde (5.5 g; 36.4 mmol) and acetic anhydride (6 mL; 63.5 mmol) where dissolved in 150 mL of propionic acid. The reaction mixture is heated to reflux (140 °C), and then 2.5 mL of pyrrole (36.0 mmol) was slowly added to the reaction mixture. The mixture continued to reflux for another 30 minutes. After reaction was completed, the reaction mixture was cooled to room temperature and filtered through a sinter funnel. The precipitate is washed with water and methanol. The remaining black powdery precipitate was dissolved in 40 mL of pyridine, and the reaction mixture was refluxed at 115 °C for 1 h. After the reaction is complete, the reaction mixture is cooled, filtered through a sintered funnel, and the precipitate was washed with acetone and left to dry for 25 min under reduced pressure. Drying afforded 1.17 g (16% yield) of a dark purple powdery product, 5,10,15,20-tetrakis(4-nitrophenyl)-21*H*,23*H*-porphyrin.

^{1}H NMR (400 MHz, DMSO-d6) δ / ppm: 8.92 (s, 8H); 8.70 (d, 8H); 8.53 (d, 8H), −2.92 (s, 2H).

IR (ATR) $\tilde{\nu}$/ cm^{-1}: 3323; 1594; 1532; 1517; 1475; 1401; 1343; 1109; 967; 863; 845.

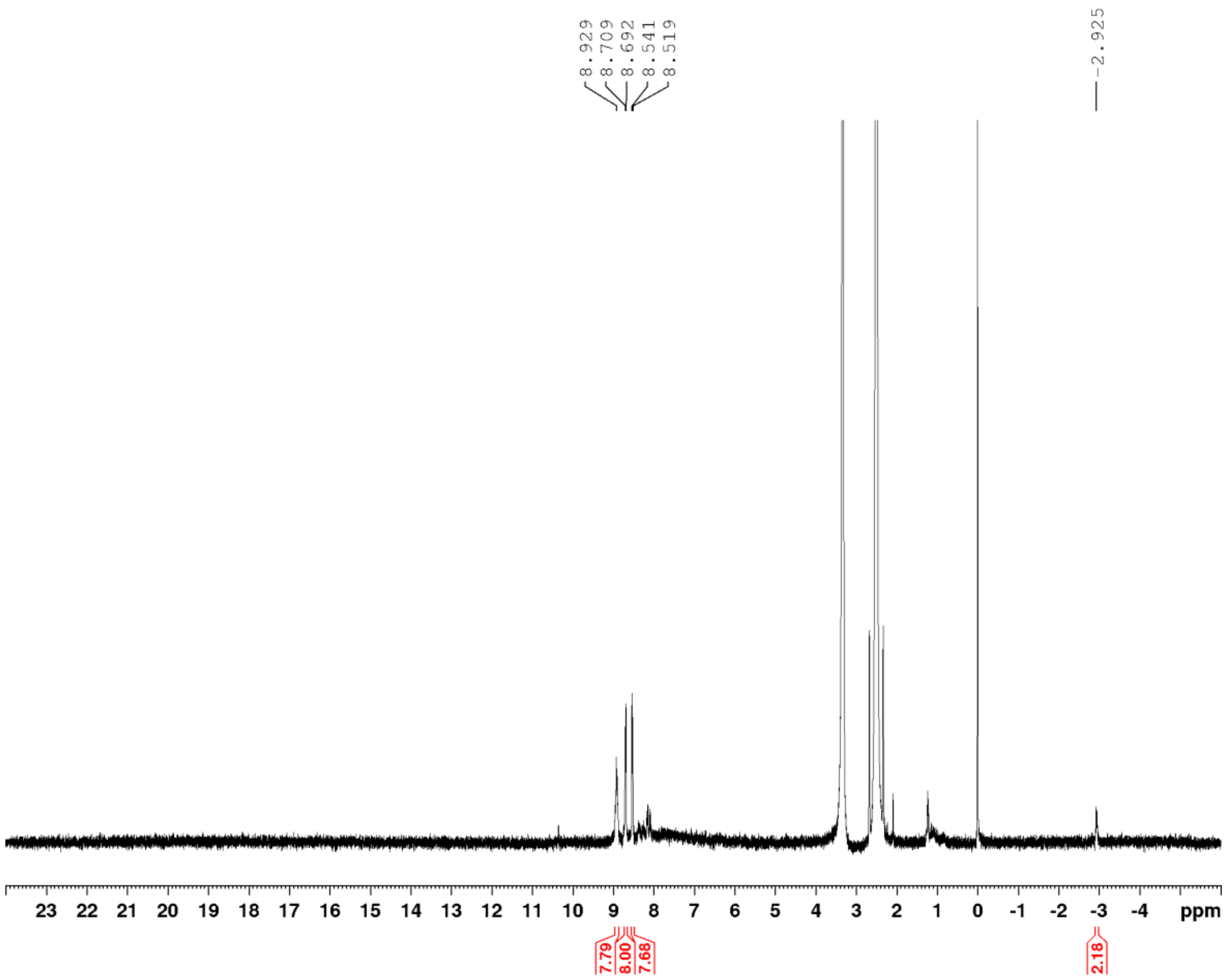

Figure S2. ^{1}H NMR (400 MHz, DMSO-d6) spectrum of TNPPR.

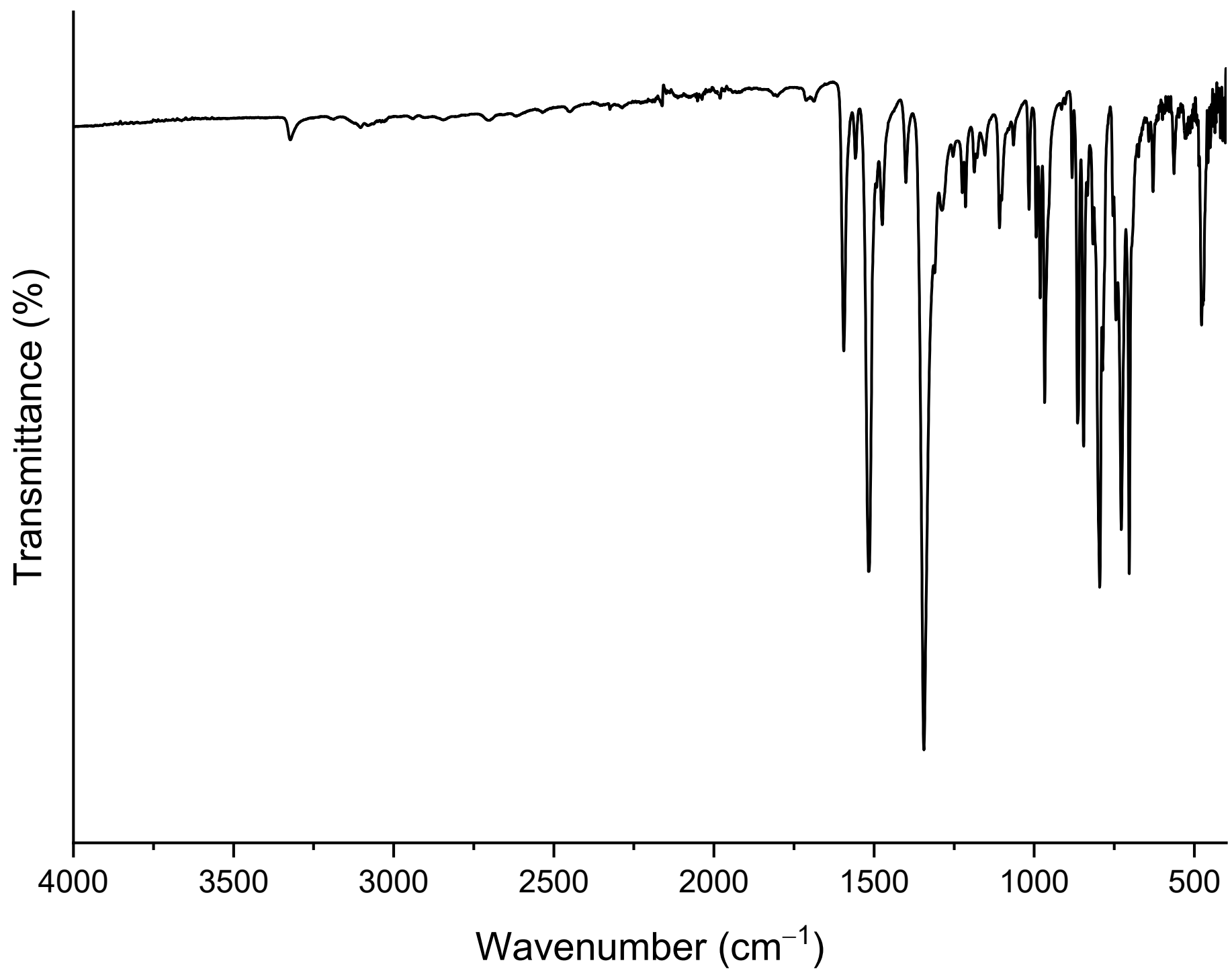

Figure S3. FTIR (ATR) spectrum of TNPPR.

1.2. Synthesis and characterization of APPs

Azo polymers APP-BP-OH, APP-AQ, APP-BP-Me and APP-Ph-Me were synthesized by a similar procedure described in the literature.[2] The syntheses were carried out according to the same general procedure, differing only in the starting amino compounds (Table S1).

TNPPR (0.29 mmol) and corresponding diamine (0.29 mmol) where added to a two -neck 100 mL round-bottom flask and dissolved in dry DMF. Reaction mixture was degassed with N_2 and set to reflux at 140 °C for 2 4h under N_2 atmosphere. After 24 h precipitate was formed and filtered. Precipitate was washed with 25 mL of H_2O, DMF, acetone, THF and MeOH. Precipitate was then transferred to Soxhlet extractor and continuously washed with acetone (24 h), $CHCl_3$ (24 h) and MeOH (24 h). After washing product was dried at 140 °C under reduced pressure for 5 h.

Table S1. List of APPs and starting amino compounds, masses and colour of APPs, and yields of reactions.

Azo polymer	Amino linker	Mass obtained (mg)	Colour of polymer	Yield of reaction (%)
**APP-BP-OH**	4,4'-diaminobiphenyl-3,3'-diol	252	Black	98
**APP-AQ**	2,6-diaminoanthraquinone	191	Dark purple	73
**APP-BP-Me**	3,3'-dimethylbiphenyl-4,4'-diamine	221	Dark purple	87
**APP-Ph-Me**	2,5-dimethylbenzene-1,4-diamine	209	Black	90

Reaction yield calculation:

The theoretical mass of the polymers was calculated as the sum of the mass of the limiting reagent (diamine) and the stochiometric equivalent of TNPPR, minus the mass of eliminated oxygen and hydrogen atoms during azo-linkage formation (34.01 g/mol per linkage).

$$Theoretical\ mass = [n(\text{diamine}) \cdot MW(\text{diamine})] + [n(\text{diamine}) * MW(\text{TNPPR})] \\ -[4 \cdot n(\text{diamine}) \cdot 34.01]$$

$$Reaction\ Yield = \frac{Mass\ obtained}{Theoretical\ mass} \cdot 100\ [\%]$$

APP-BP-OH

IR (ATR) $\tilde{\nu}$ / cm^{-1}: 3286; 3037; 1596; 1515; 1466; 1396; 1346; 1282; 1177; 964; 793; 708.

^{13}C CP/MAS NMR (400 MHz) δ / ppm: 151.4; 144.3; 131.1; 118.8; 113,6.

Elemental analysis: 69.28 %C (calc. 76.52); 4.05 %H (calc. 3.90); 14.24 %N (calc. 15.94).

APP-AQ

IR (ATR) $\tilde{\nu}$ / cm^{-1}: 3291; 3023; 2336; 1588; 1515; 1465; 1400; 1343; 1343; 1294; 964; 792; 709.

^{13}C CP/MAS NMR (400 MHz) δ / ppm: 180.1, 151.7, 144.7, 130.7, 118.9, 113.8.

Elemental analysis: 73.40 %C (calc. 77.32); 5.25 %H (calc. 3.58); 15.12 %N (calc. 15.55).

APP-BP-Me

IR (ATR) $\tilde{\nu}$ / cm^{-1}: 3309; 3026; 2881; 1691; 1596; 1508; 1470; 1395; 1342; 1277; 1220; 1178; 965; 793; 707.

^{13}C CP/MAS NMR (400 MHz) δ / ppm: 152.0; 144.2; 130.7; 118,7; 113,9; 33,5; 16.5.

Elemental analysis: 68.76 %C (calc. 79.60); 4.55 %H (calc. 4.38); 14.92 %N (calc. 16.01).

APP-Ph-Me

IR (ATR) $\tilde{\nu}$ / cm^{-1}: 3321; 3028; 2885; 1595; 1519; 1471; 1402; 1347; 1182; 967; 798; 728.

^{13}C CP/MAS NMR (400 MHz) δ / ppm: 153.3; 144.3; 131.0, 119.1, 15.2.

Elemental analysis: 67.13 %C (calc. 78.20); 3.80 %H (calc. 4.29); 14.30 %N (calc. 17.53).

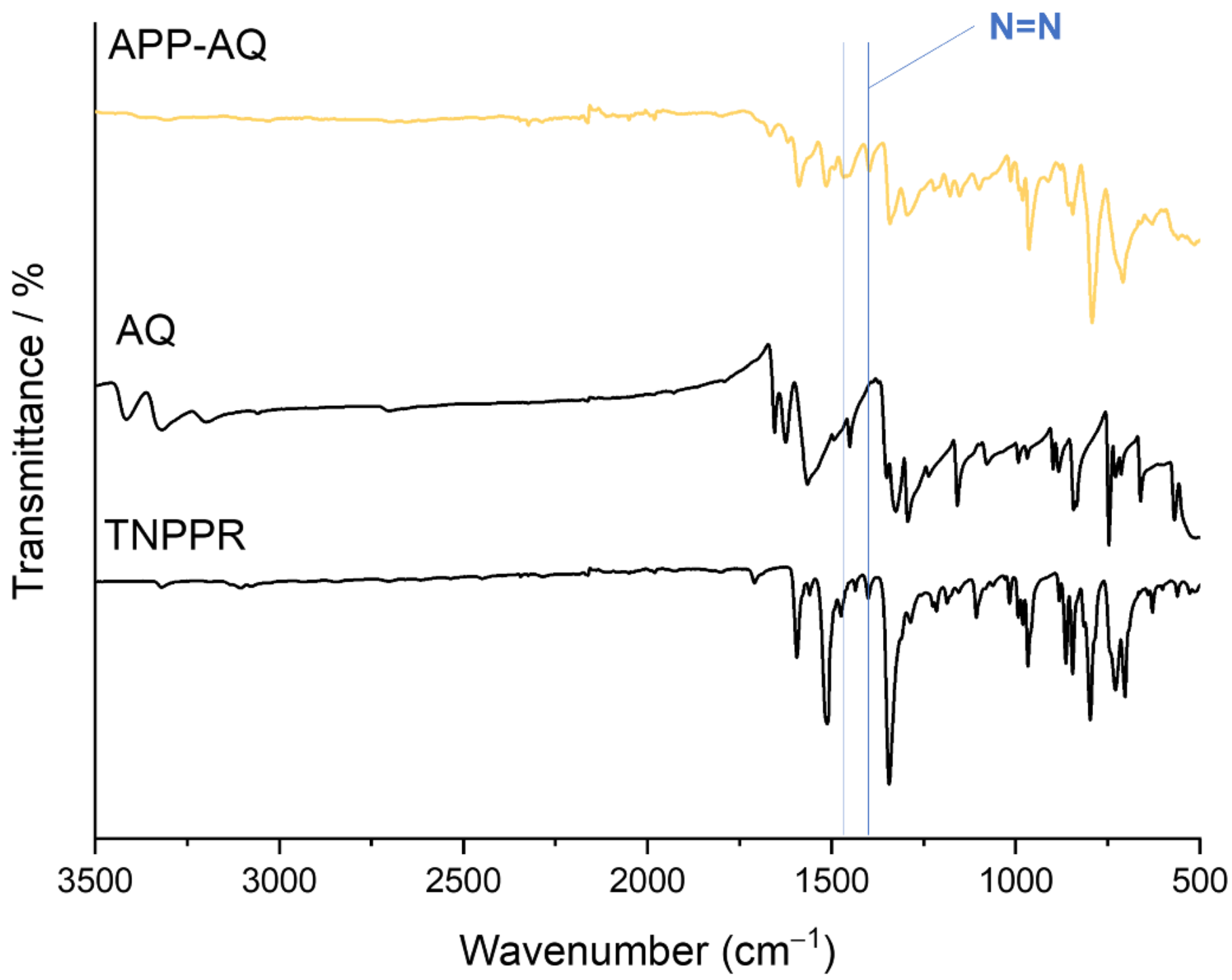

Figure S4. Comparison of FT-IR spectra of APP-AQ and starting compounds (AQ and TNPPR).

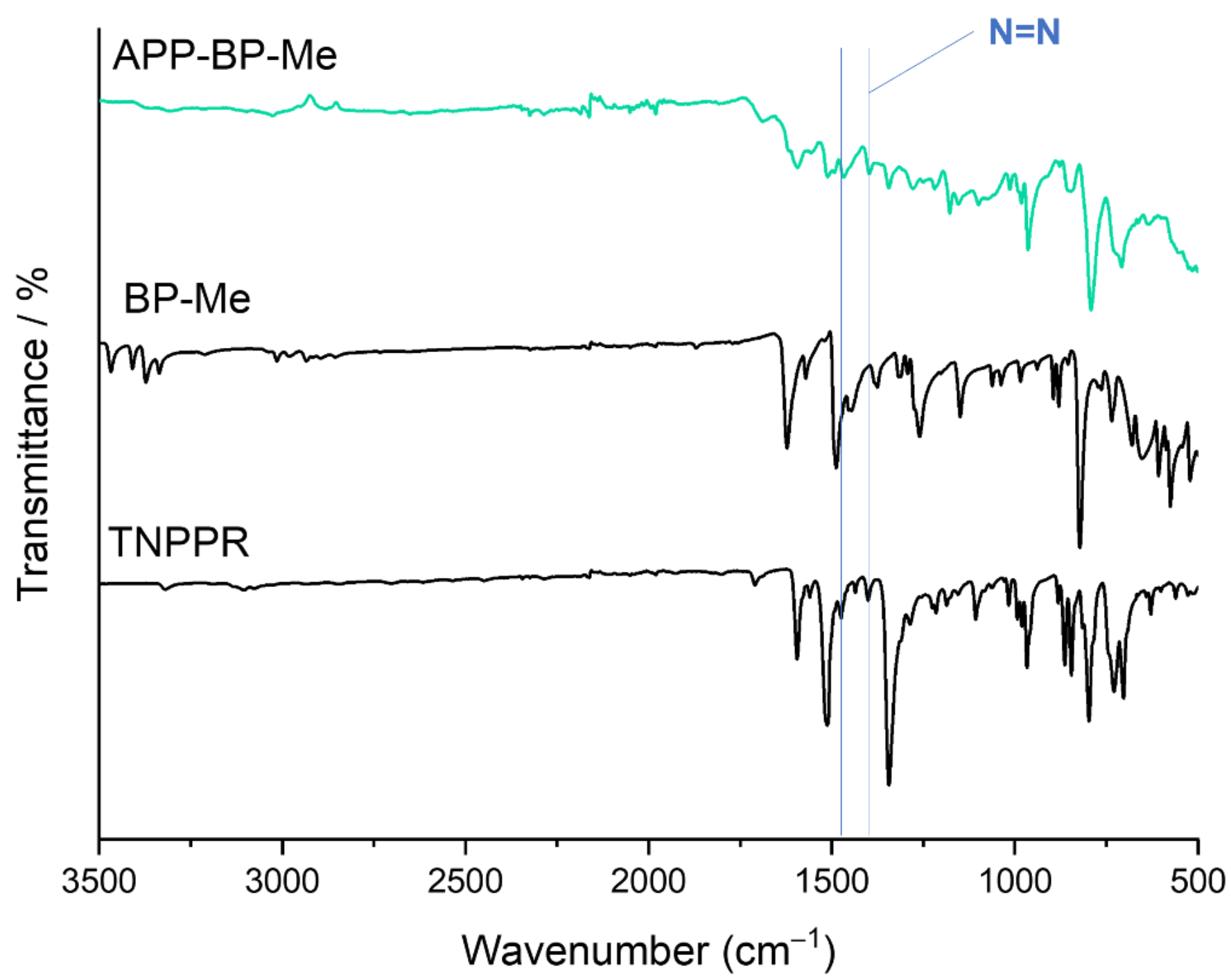

Figure S5. Comparison of FT-IR spectra of APP-BP-Me and starting compounds (BP-Me and TNPPR).

1.3. Thermogravimetric analysis

APP-Ph-Me and APP-AQ have similar decomposition profiles. Both samples are stable up to ~240 °C when a slow decomposition event starts and the decomposition rate gradually increases reaching a local maximum at ~400 °C, then a small decrease in the decomposition rate is observed until 430 °C and both samples lose around 4.8 % of their initial weight. At this point the second thermal event starts and a significant increase in the rate of decomposition is observed reaching a maximum at ~510 °C. Sample APP-Ph-Me shows a slightly higher decomposition rate with a plateau from 650 to 750 °C and a higher loss in weight, 24.5 % of its initial weight (29.3 % total weight loss) than sample APP-AQ which shows a continuous decrease in decomposition rate until the end of the isothermal step at 800 °C and losses 20.2 % of its initial weight (25.0 % total weight loss).

APP-BP-Me and APP-BP-OH display lower thermal stability and have a less pronounced first decomposition event starting at ~210 °C (APP-BP-OH) or ~230 °C (APP-BP-Me) and at 430 °C both samples show a great increase in the decomposition rate as the second thermal event starts. Sample APP-BP-Me loses 4.0 % of its initial weight by 430 °C and shows a maximum during the second decomposition step at a lower temperature (< 500 °C) than all the other samples, it loses an additional 21.8 % of its initial weight by 800 °C (25.8 % total weight loss). Sample APP-BP-OH shows the highest decrease in weight during both decomposition events and loses 7.0 % of its initial weight by 430 °C, it shows a maximum during the second decomposition step at ~510 °C and loses 26.2 % of its initial weight by 800 °C (33.2 % total weight loss).

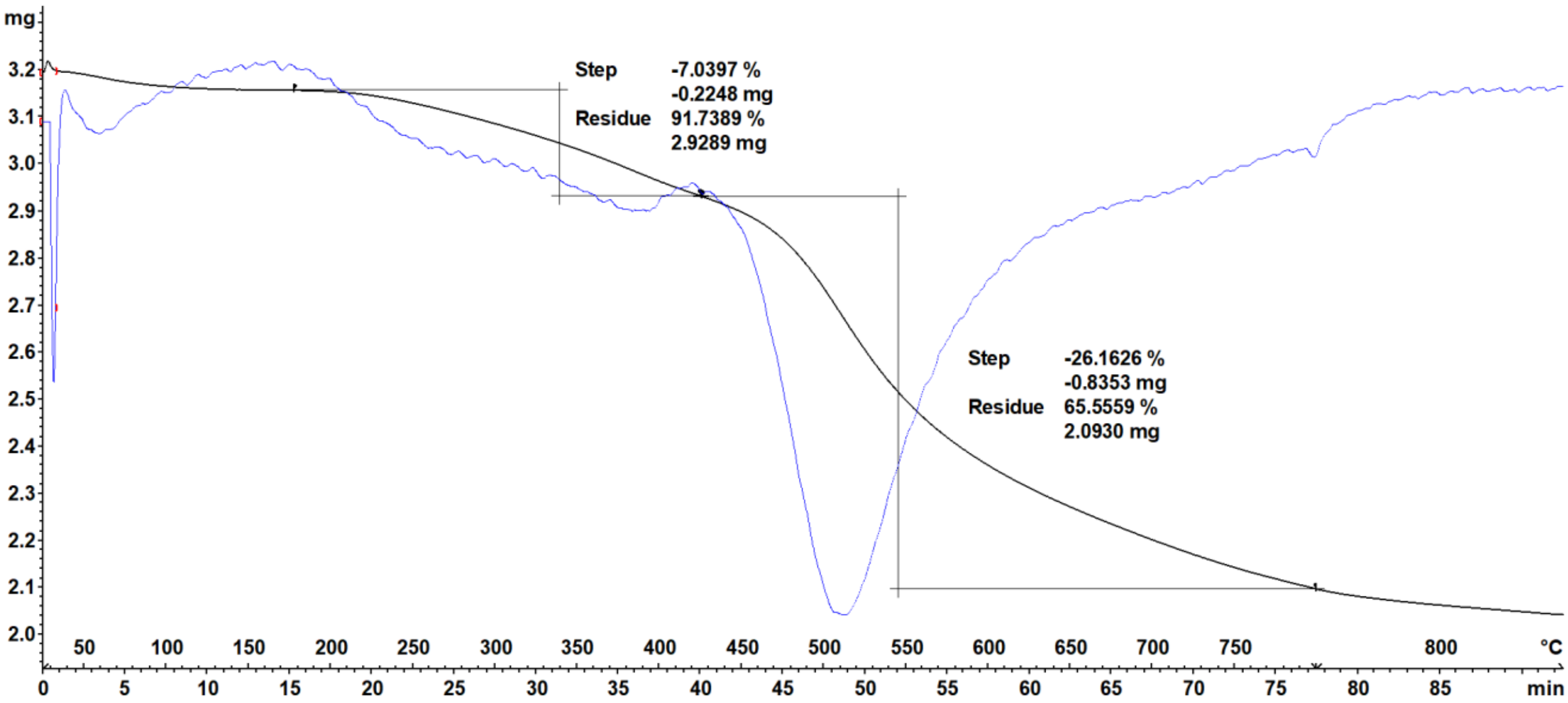

Figure S6. TGA (black), 1st derivative of weight (blue) of APP-BP-OH.

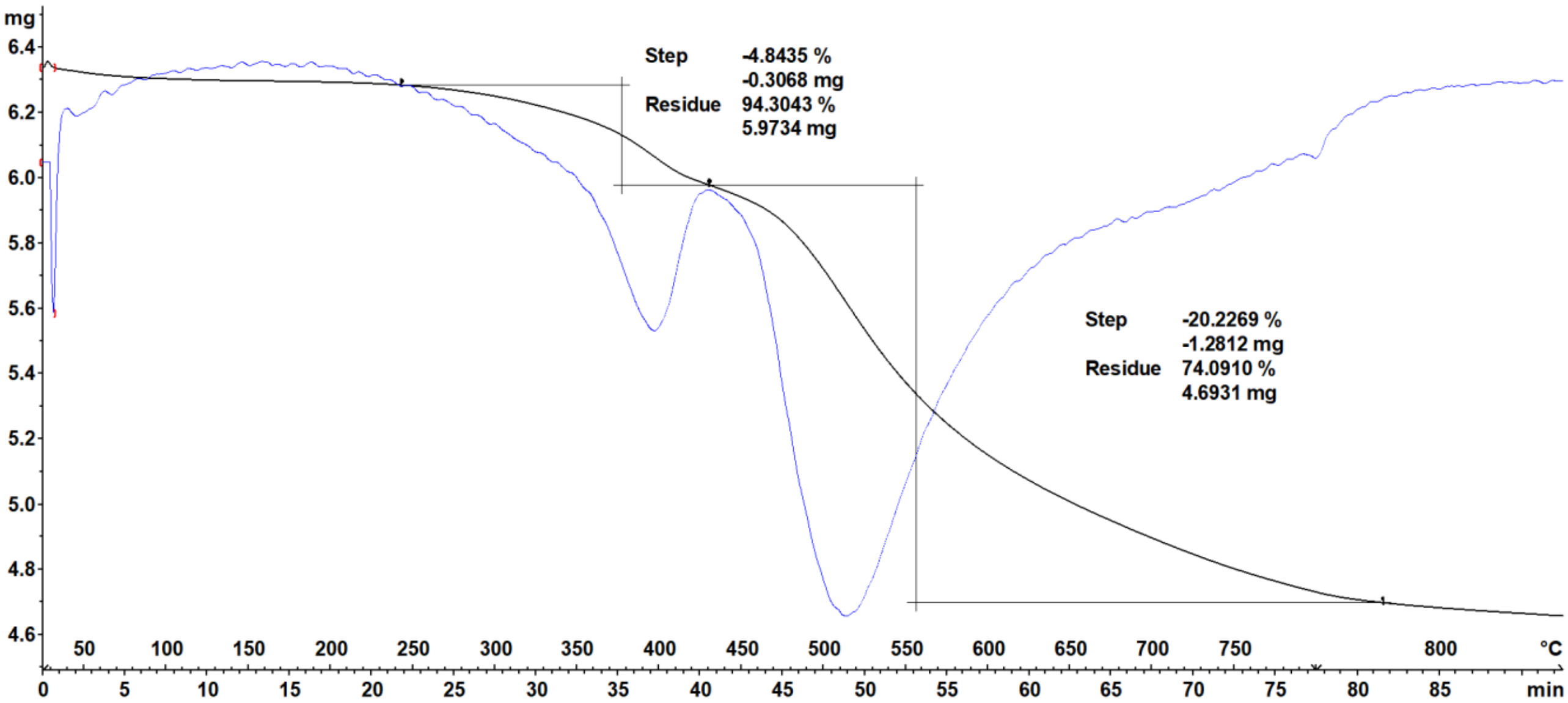

Figure S7. TGA (black), 1st derivative of weight (blue) of APP-AQ.

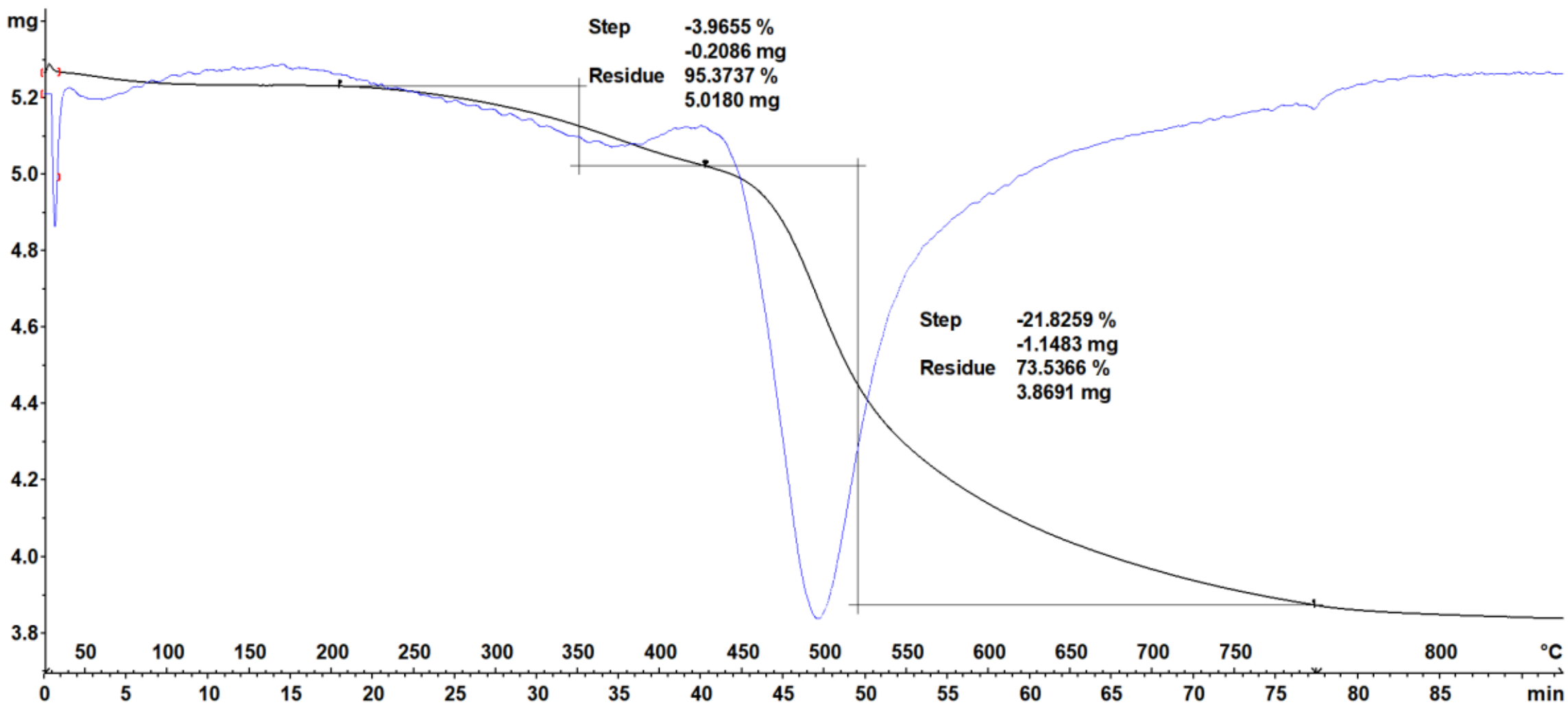

Figure S8. TGA (black), 1st derivative of weight (blue) of APP-BP-Me.

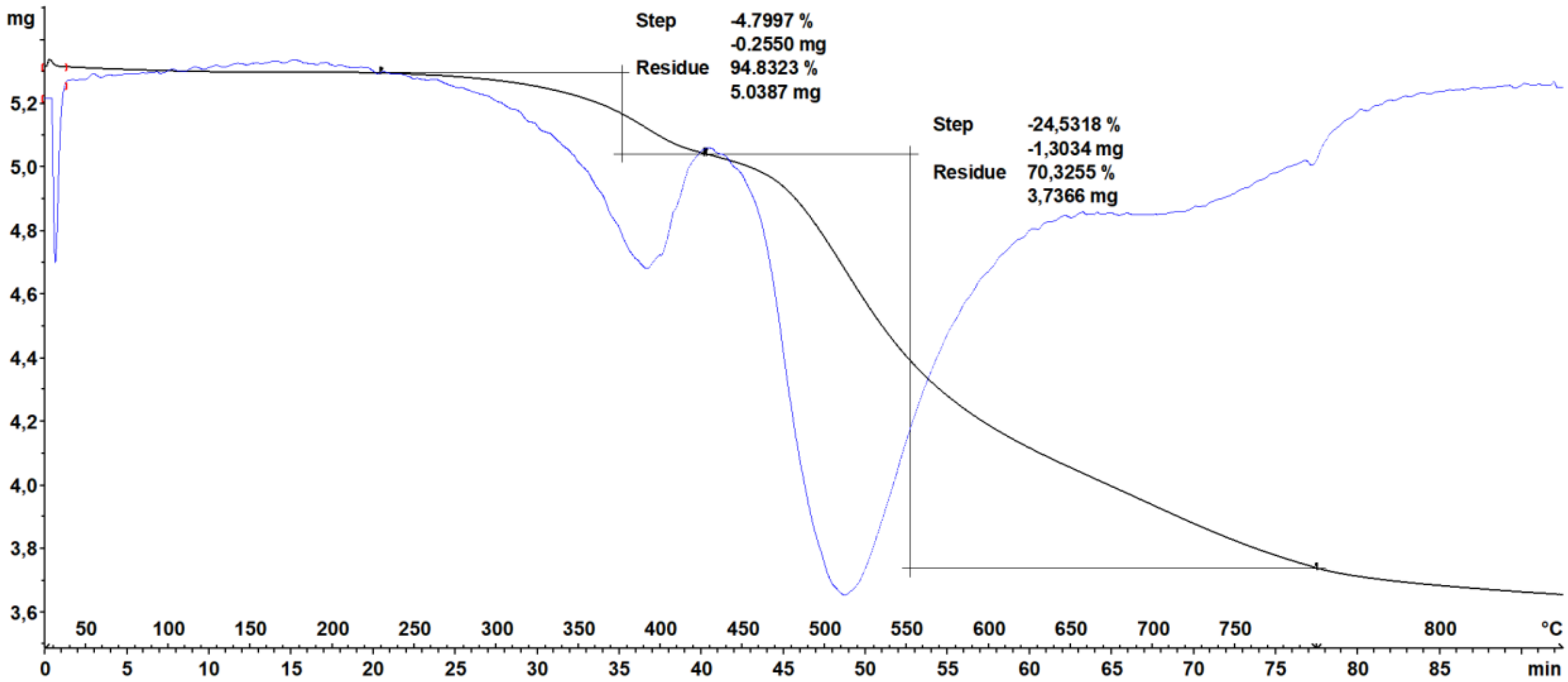

Figure S9. TGA (black), 1st derivative of weight (blue) of APP-Ph-Me.

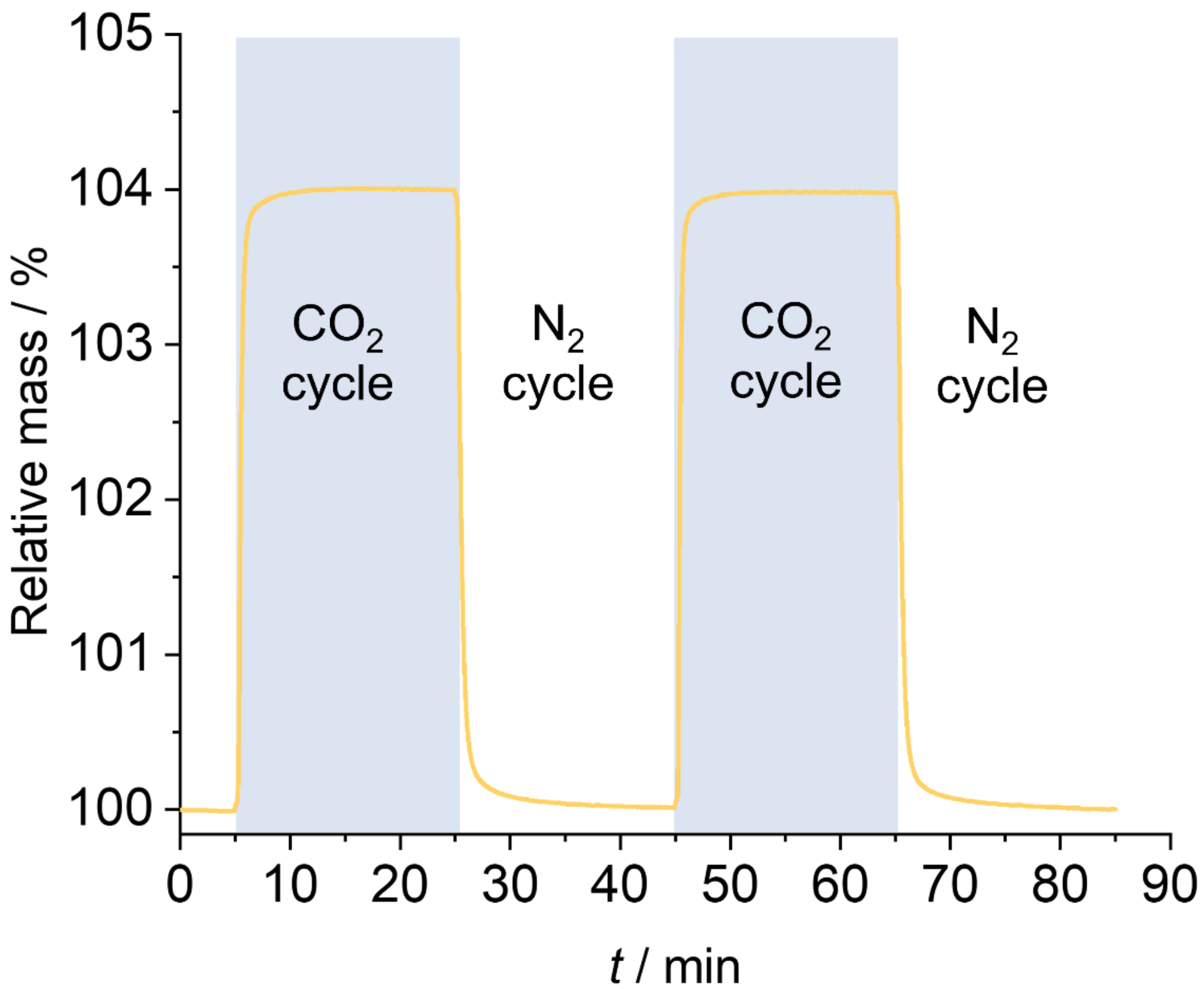

Figure S10. Thermogravimetric CO_2 adsorption and desorption profiles of APP-AQ at approximately 30 °C (303 K).

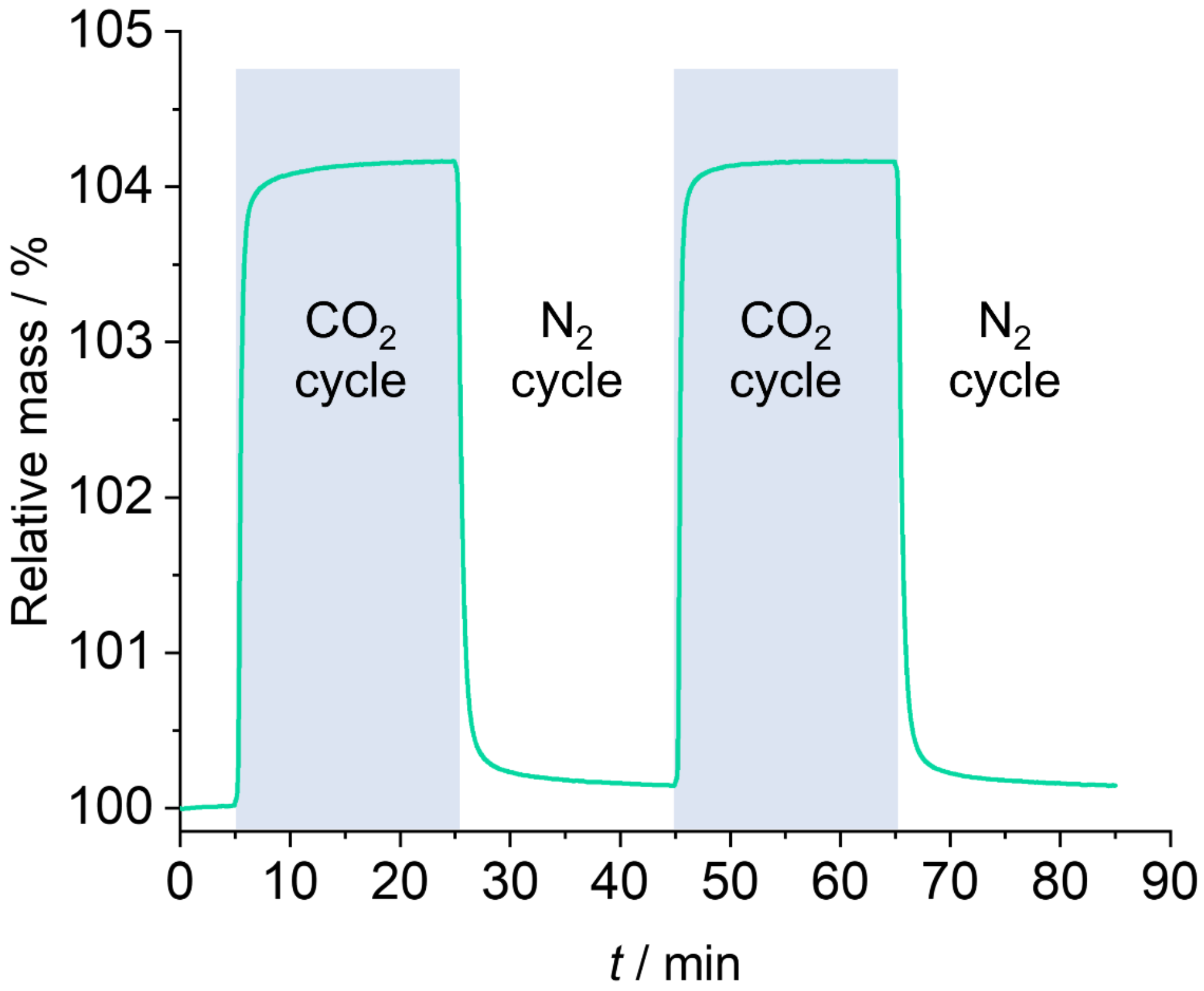

Figure S11. Thermogravimetric CO_2 adsorption and desorption profiles of APP-BP-Me at approximately 30 °C (303 K).

2. Computational studies of APPs model systems

2.1. Optimized geometries of APPs

Table S2. Unit cell parameters (*a*, *b*, *c*, α, β and γ) of the optimized APPs geometries (PBE-D3/pob-TZVP-rev2). The labels of the layer group and space groups used in CRYSTAL23.

Compound	Space Group	CRYSTAL23 label	*a* (Å)	*B* (Å)	*C* (Å)	*α* (°)	*β* (°)	*γ* (°)
APP-BP-OH	*P m n a*	53	43.8956	38.5176	3.7064	90	90	90
APP-AQ	$P\,2_1/m$	11	36.4450	44.3738	3.7799	90	100.32	90
APP-BP-Me	*P m n a*	53	46.1459	36.1217	3.7258	90	90	90
APP-Ph-Me	C 2/m	12	32.6650	36.4876	3.8275	90	94.01	90

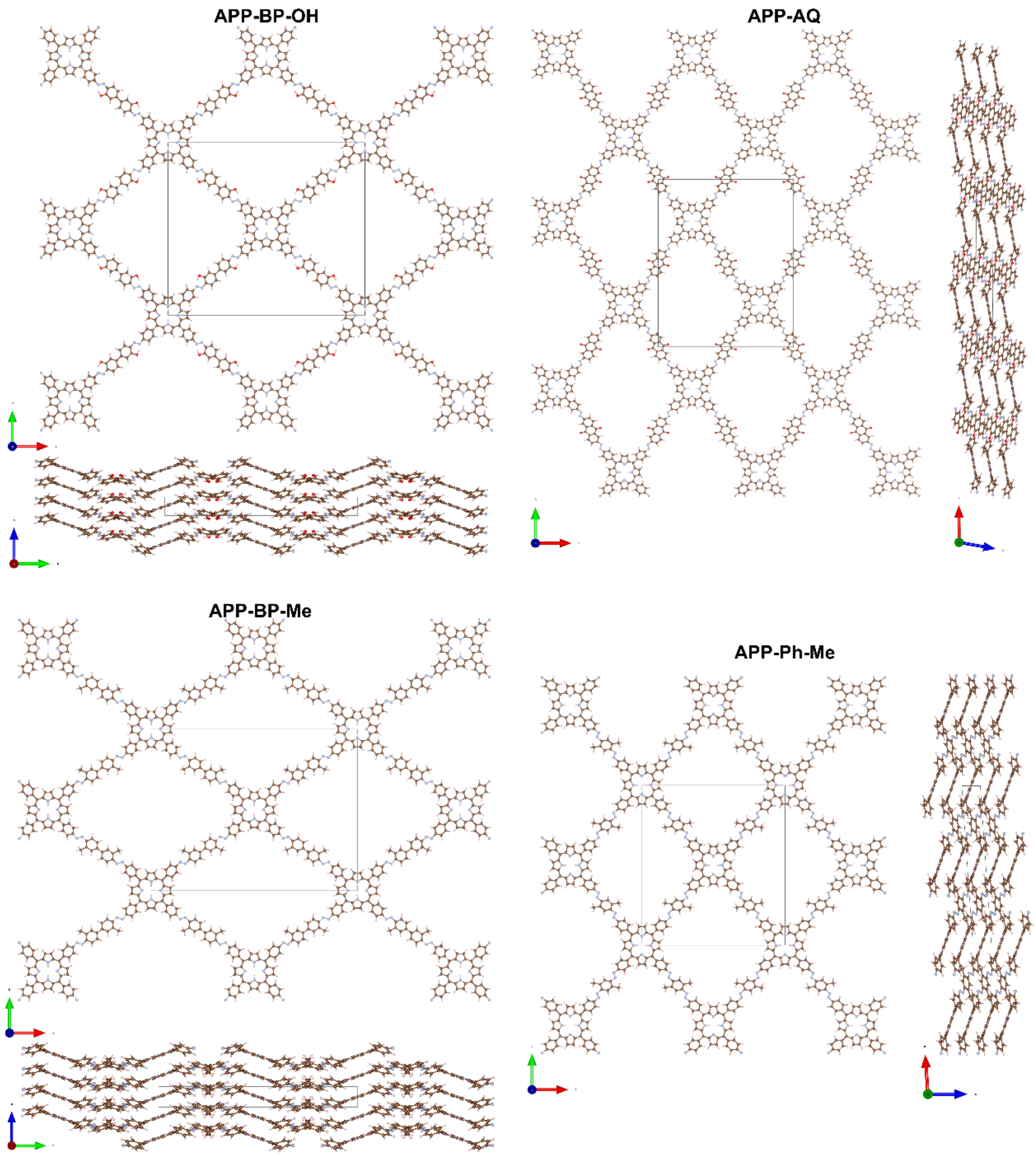

Figure S12. Optimized geometries (PBE-D3/pob2-TZVP-rev2) of APPs with eclipsed (AA-stacked) 2D layers.

2.2. Electrostatic potential maps

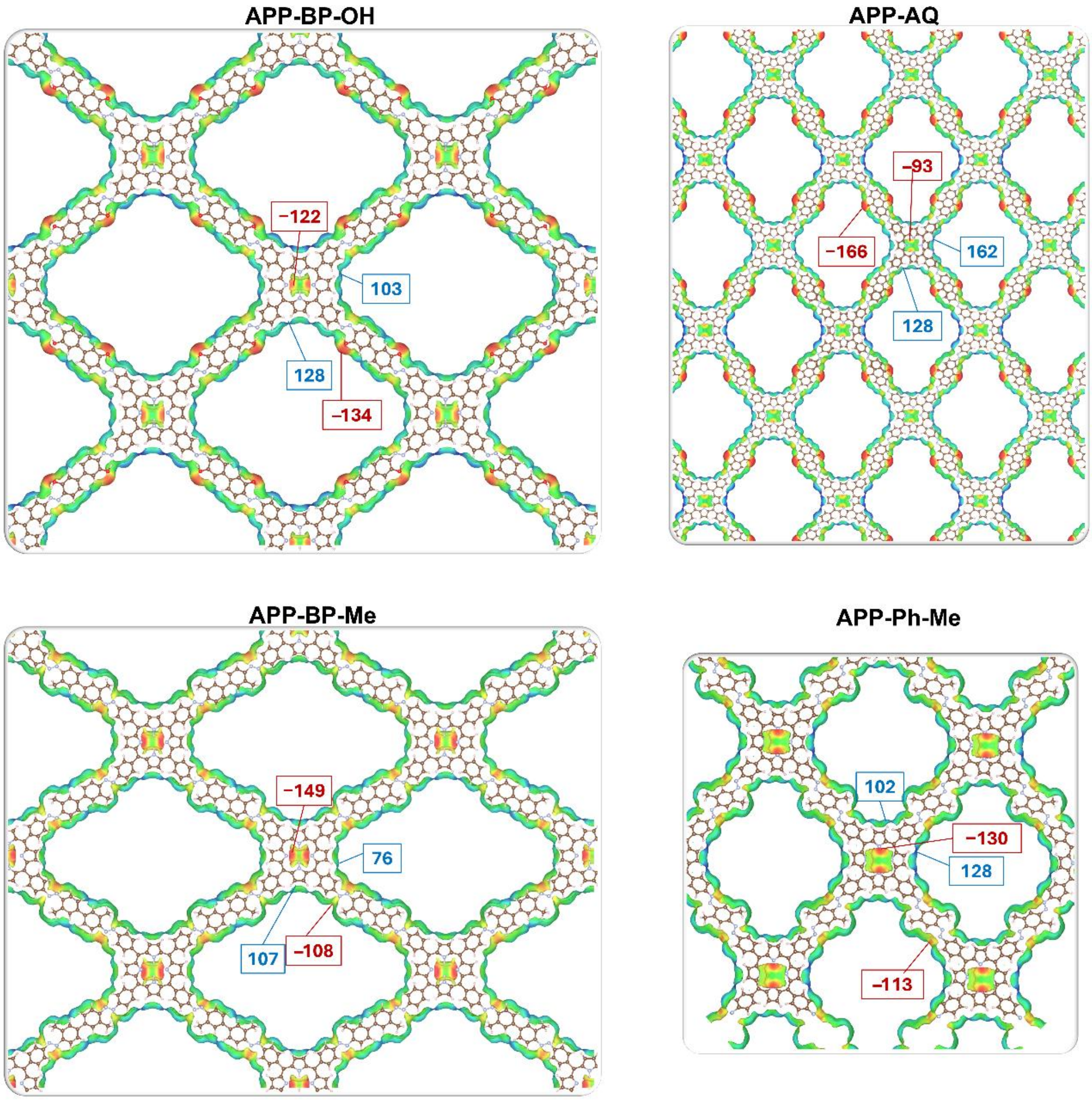

Figure S13. Electrostatic potential maps of APPs. Geometries optimized at the PBE-D3/pob-TZVP-rev2 level of theory. The calculated ESP values are expressed in kJ mol^{-1} e^{-1}.

2.3. Calculated adsorption isotherms and density plots

Table S3. Calculated framework properties of APPs including the size of the simulation box used in the GCMC calculations, framework density, available pore volume and nitrogen accessible surface area.

Compound	GCMC simulation box	Framework density (g cm^{-3})	Available pore volume (cm^3 g^{-1})	Average surface area (m^2 g^{-1})
APP-BP-OH	2x2x8	0.578	0.782	1750.18
APP-AQ	2x2x8	0.575	0.785	1790.46
APP-BP-Me	2x2x8	0.579	0.734	1804.05
APP-Ph-Me	2x2x8	0.680	0.520	1659.59

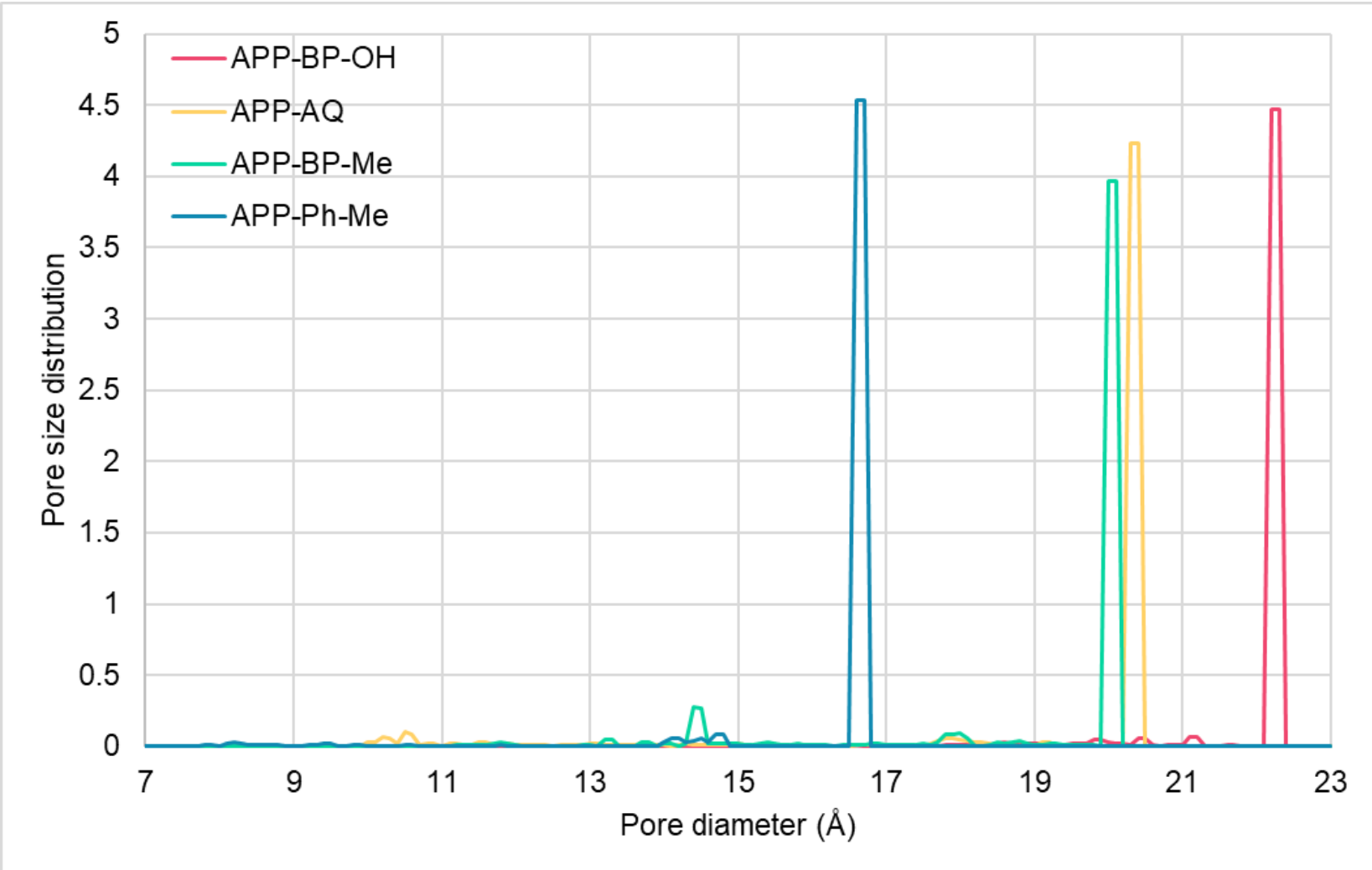

Figure S14. Pore size distribution of APPs.

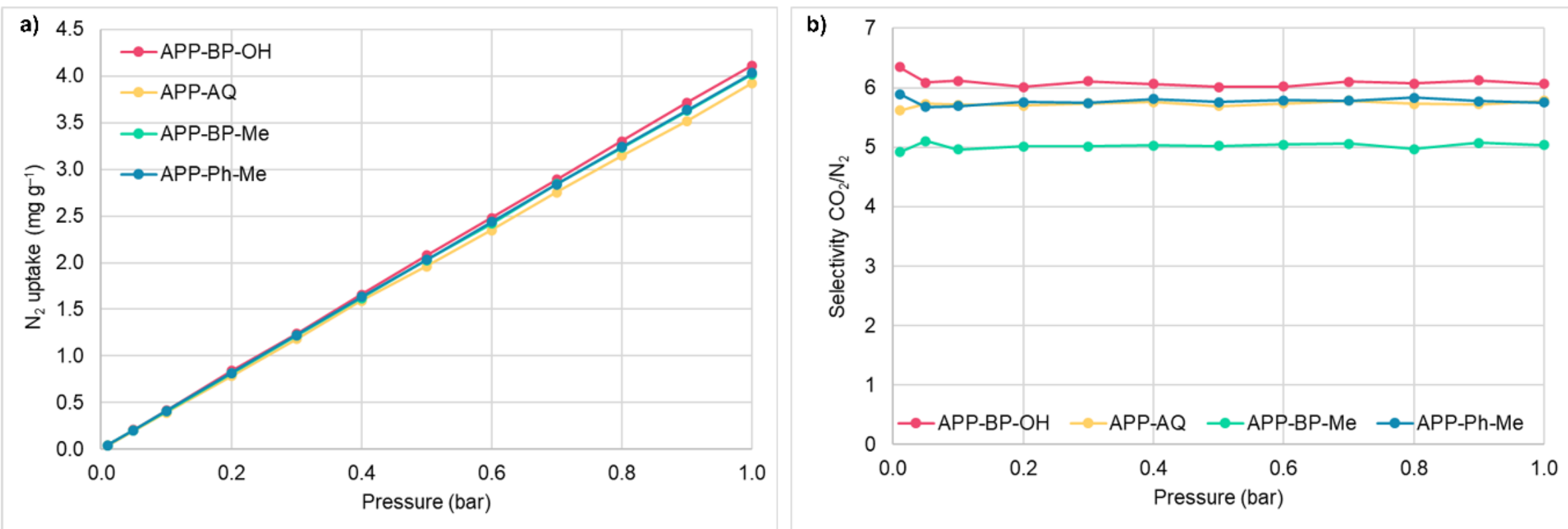

Figure S15. a) N_2 adsorption isotherms of APPs at 298 K. b) CO_2/N_2 selectivities of APPs for binary mixtures (15 : 85 molar ratio) at 298 K.

Table S4. Calculated adsorption uptake of N_2 at pressure of 1 bar and 298 K. Average CO_2/N_2 selectivities of APPs for binary mixtures (15 : 85 molar ratio) at 298 K.

Compound	N_2 uptake (mg g^{-1})	Selectivity CO_2/N_2
APP-BP-OH	4.11	6.10
APP-AQ	3.92	5.73
APP-BP-Me	4.02	5.02
APP-Ph-Me	4.03	5.77

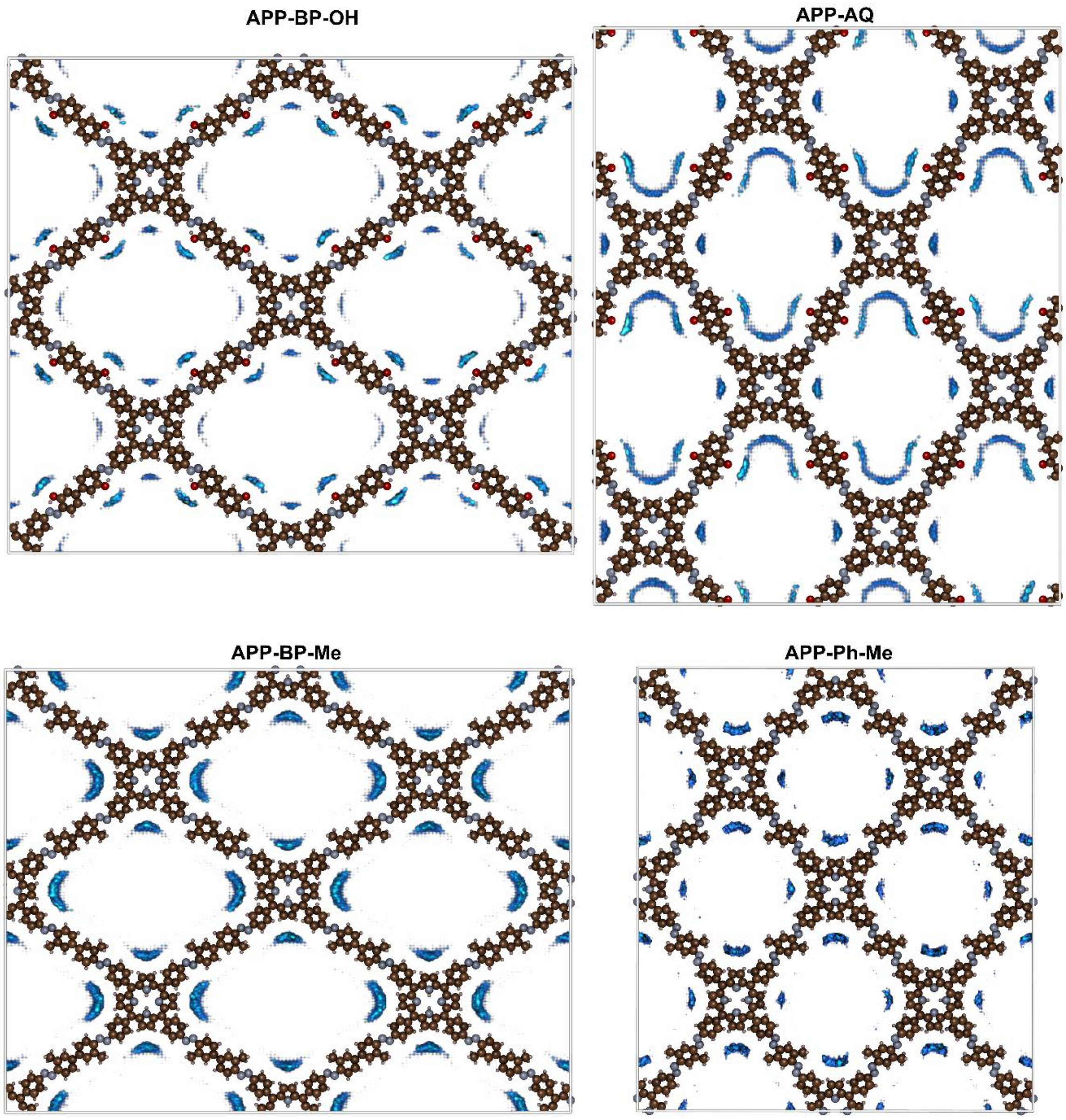

Figure S16. Density plots showing the average distribution of adsorbed CO_2 molecules of APPs at 298 K and 1 bar.

2.4. Stacking modes of APP-BP-OH

Table S5. Unit cell parameters (*a*, *b*, *c*, α, β and γ) of the optimized configurations of APP-BP-OH (PBE-D3/pob-TZVP-rev2). Energies are reported relative to the energy of the AA (eclipsed) APP-BP-OH configuration.

Stacking mode	Space Group	***a*** **(Å)**	***B*** **(Å)**	***C*** **(Å)**	***α*** **(°)**	***β*** **(°)**	***γ*** **(°)**	***E*** **(kJ mol⁻¹)**
AA (eclipsed)	*P m n a*	43.8956	38.5176	3.7064	90	90	90	0.0
AA' (serrated)	*P b c n*	45.8668	33.6143	8.1569	90	90	90	489.8
AB (staggered)	*P m n a*	44.9025	37.3227	6.2867	90	90	90	432.2

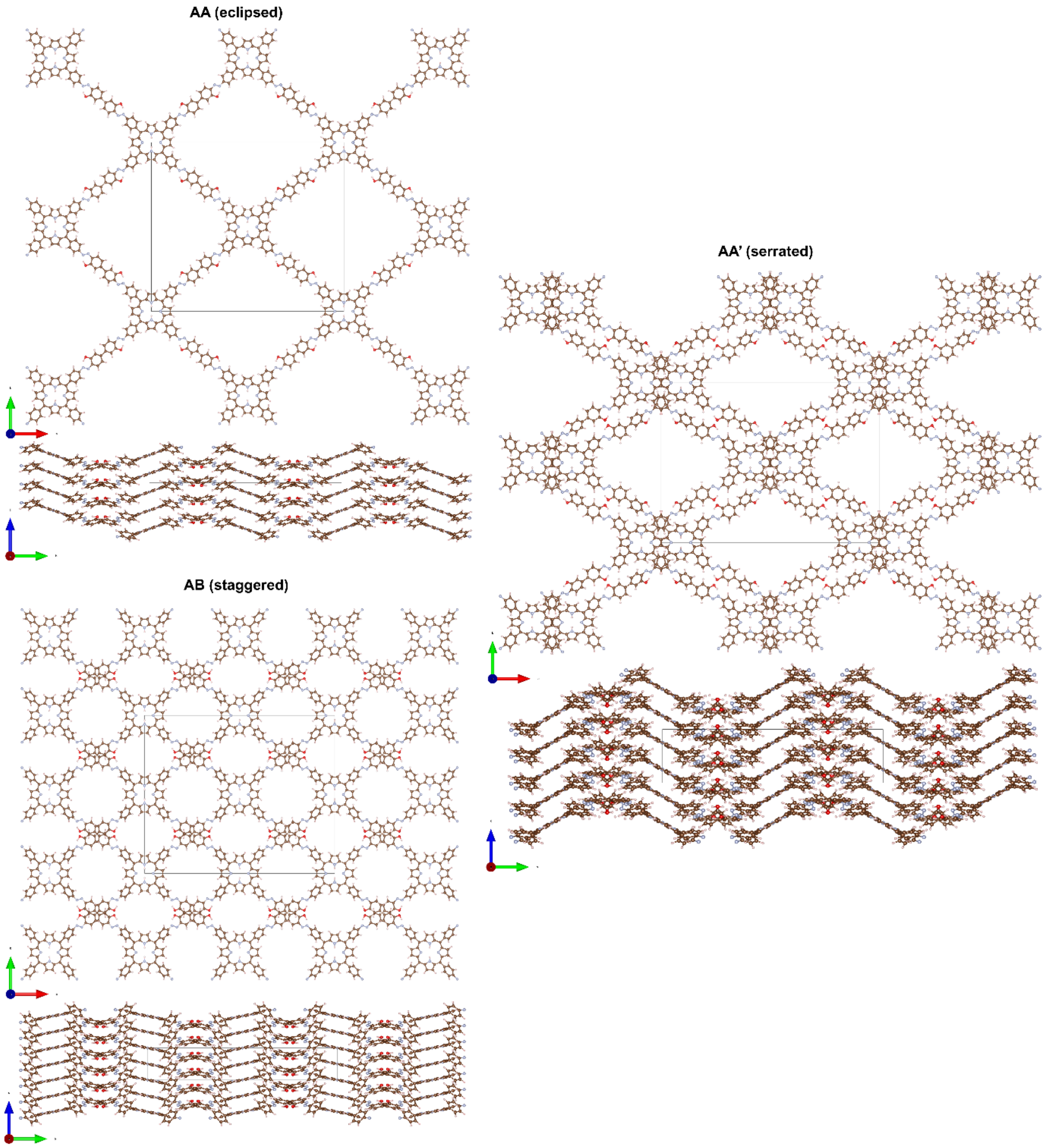

Figure S17. Optimized geometries (PBE-D3/pob-TZVP-rev2) of different 2D layer-stacking modes of APP-BP-OH shown along cell vectors *a* and *c*.

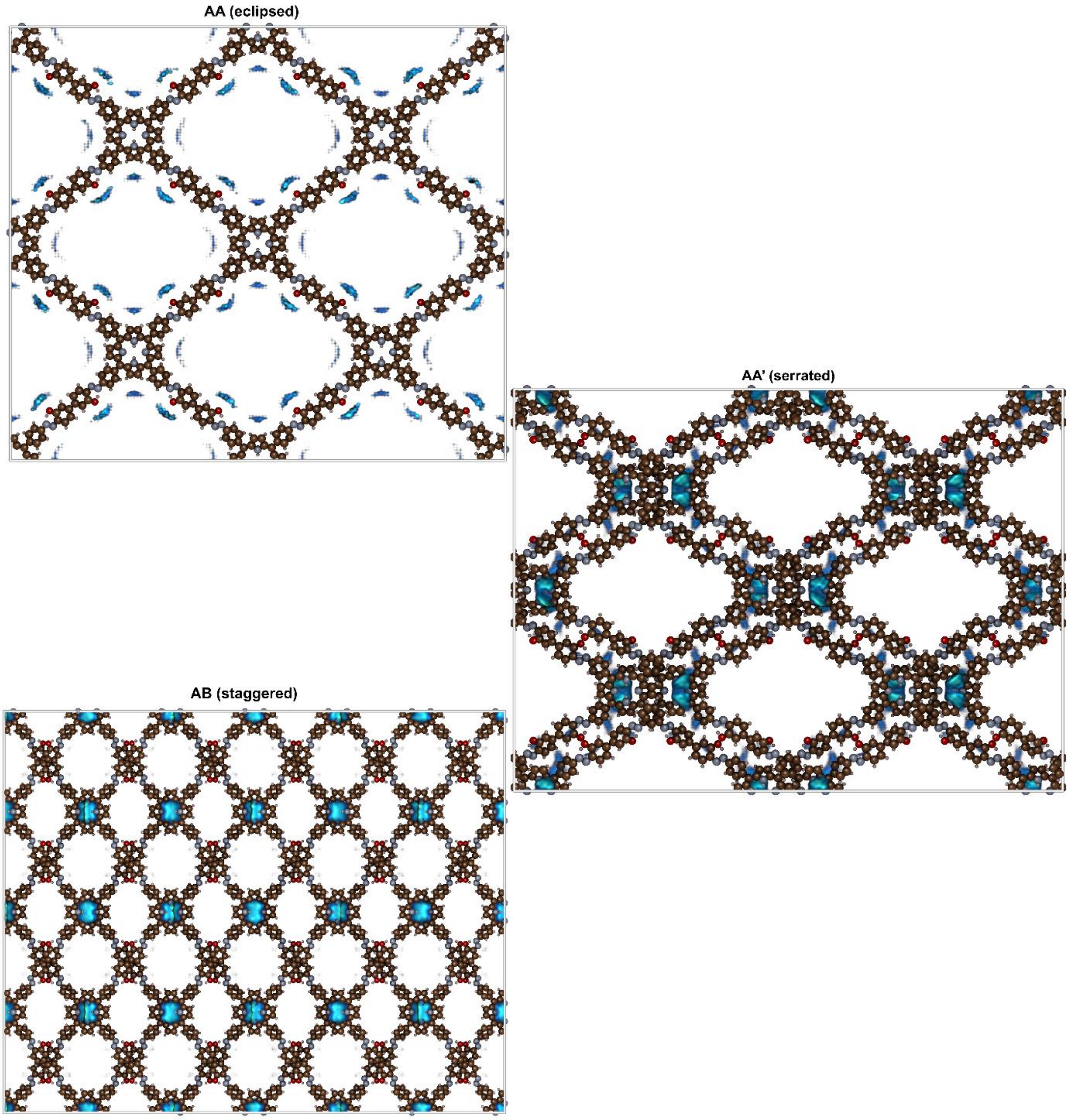

Figure S18. Density plots showing the average distribution of adsorbed CO_2 molecules of different 2D layer-stacking modes of APP-BP-OH at 298 K and 1 bar.

Table S6. Calculated framework properties of different 2D layer-stacking modes of APP-BP-OH including framework density, available pore volume, nitrogen surface area and the size of the simulation box used in the GCMC calculations.

Compound	GCMC simulation box	Framework density (g cm^{-3})	Available pore volume (cm^3 g^{-1})	Average surface area (m^2 g^{-1})	CO_2 uptake (mg g^{-1})
AA (eclipsed)	2x2x8	0.578	0.782	1750.18	24.9
AA' (serrated)	2x2x8	0.576	0.451	2716.34	212.8
AB (staggered)	2x2x8	0.688	0.241	1893.85	85.2

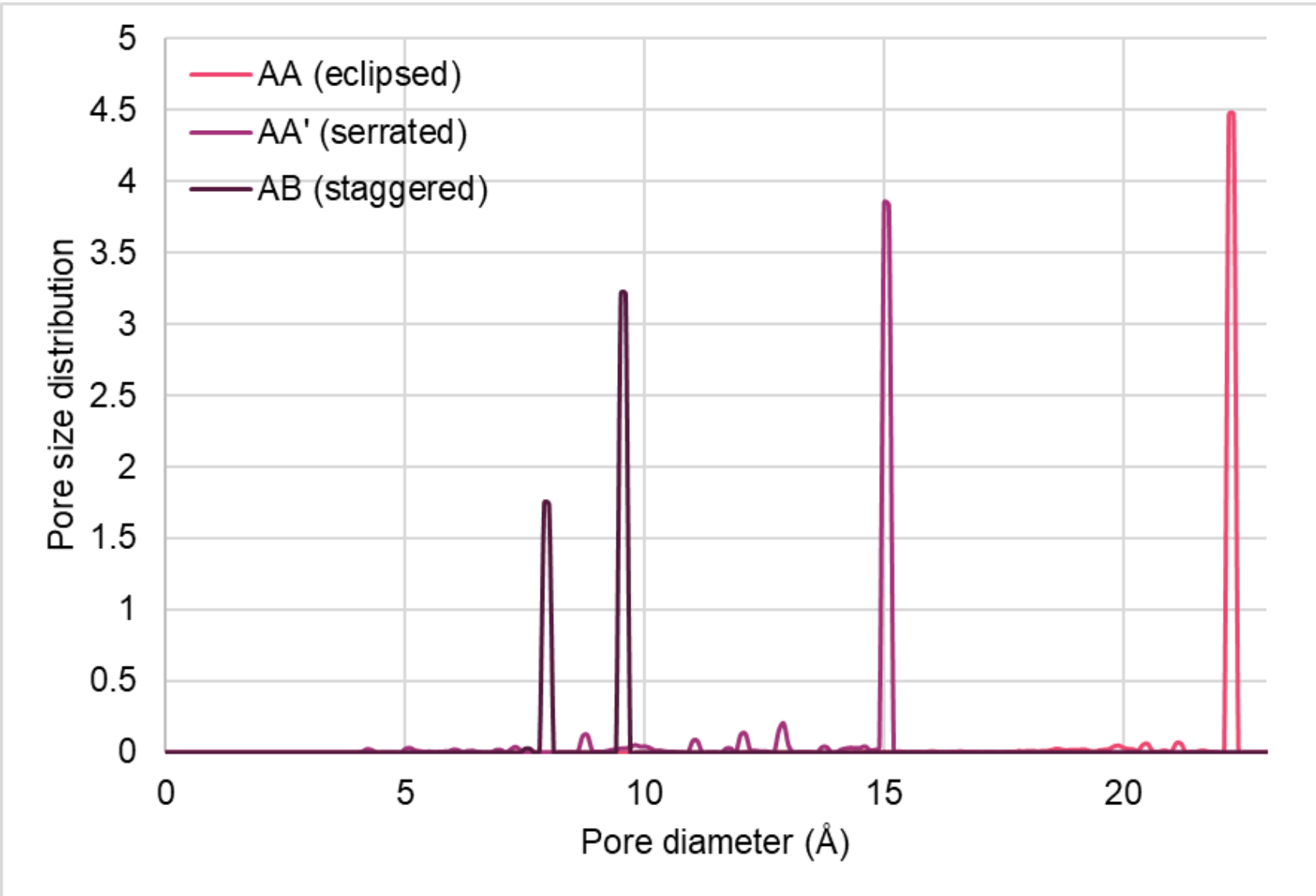

Figure S19. Pore size distribution of 2D layer-stacking modes of APP-BP-OH.

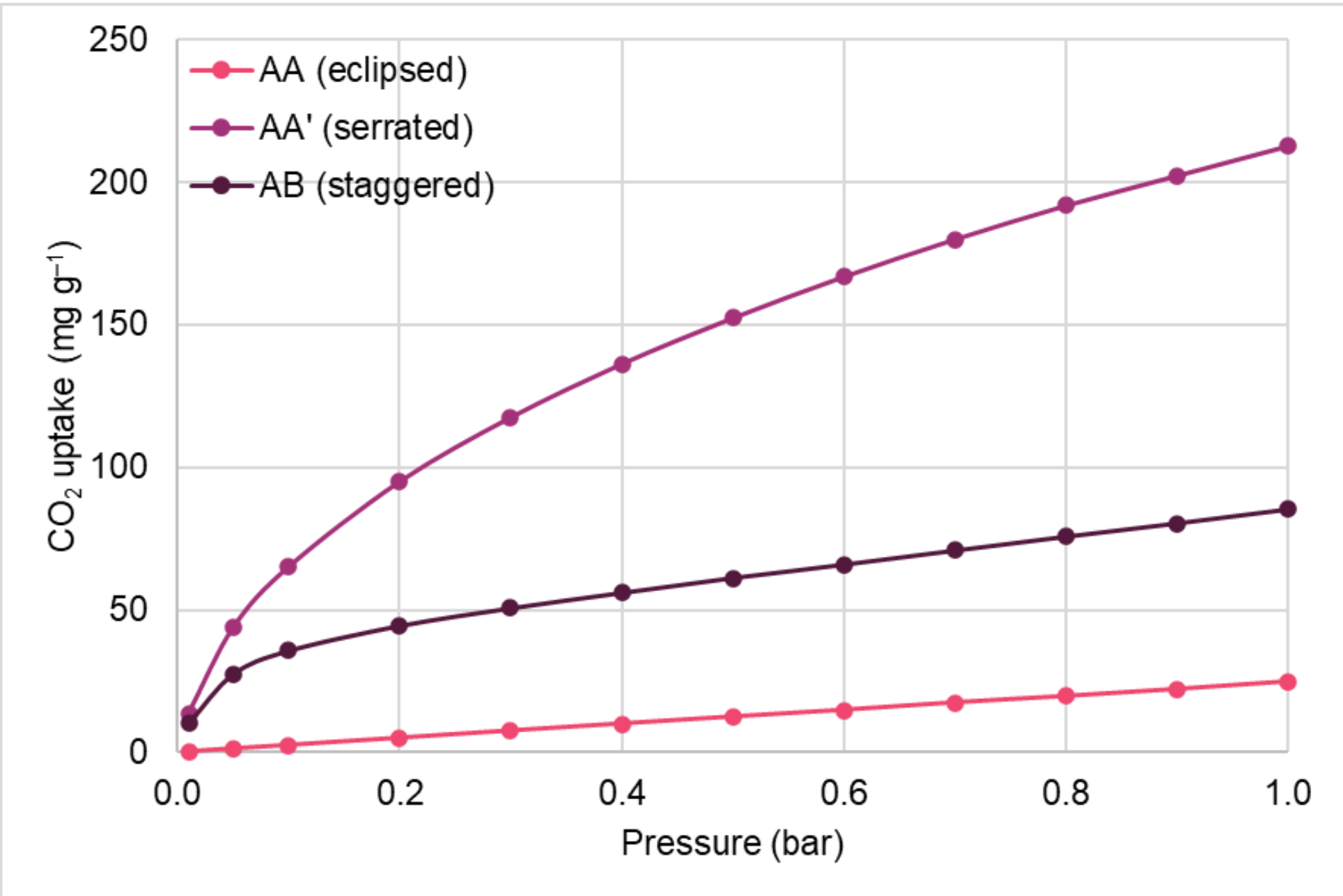

Figure S20. CO_2 adsorption isotherms of 2D layer-stacking modes of APP-BP-OH at 298 K.

2.5. Interaction energies calculated for CO_2···APPs complexes

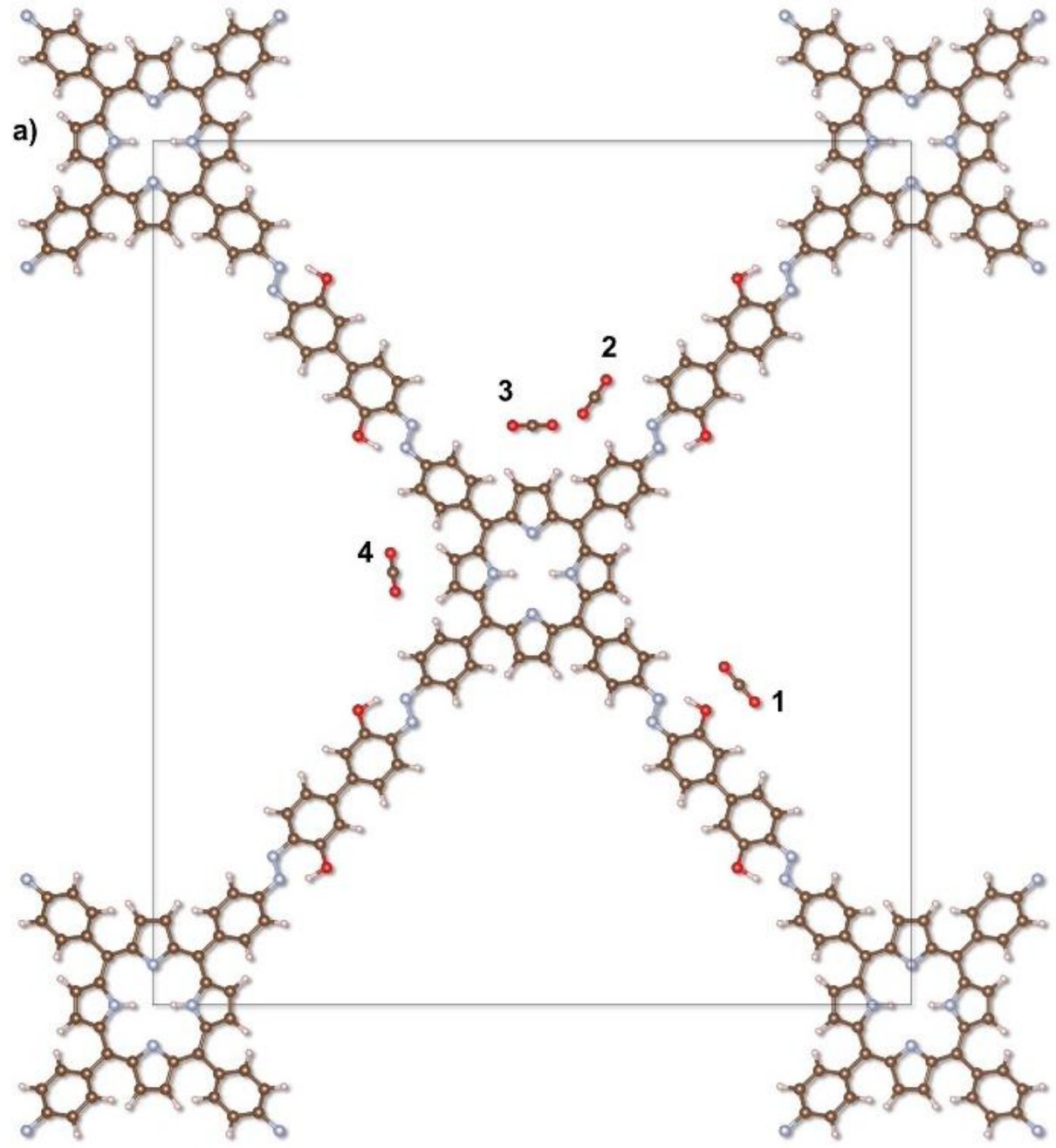

b)

CO_2···APP-BP-OH complex	Relative energy (kJ mol^{-1})	Interaction energy (kJ mol^{-1})
1	0.00	–31.01
3	1.06	–19.47
4	1.35	–18.52
5	3.50	–18.03

Figure S21. a) Overlay of optimized geometries (PBE-D3/pob-TZVP-rev2) for the selected CO_2···APP-BP-OH complexes, illustrating different CO_2 binding positions within the framework. All complexes are superimposed to highlight structural variations in CO_2 adsorption sites. b) Table summarizing the relative energies (in kJ mol^{-1}) of the complexes along with BSSE-corrected interaction energies between a single CO_2 and the APP-BP-OH framework.

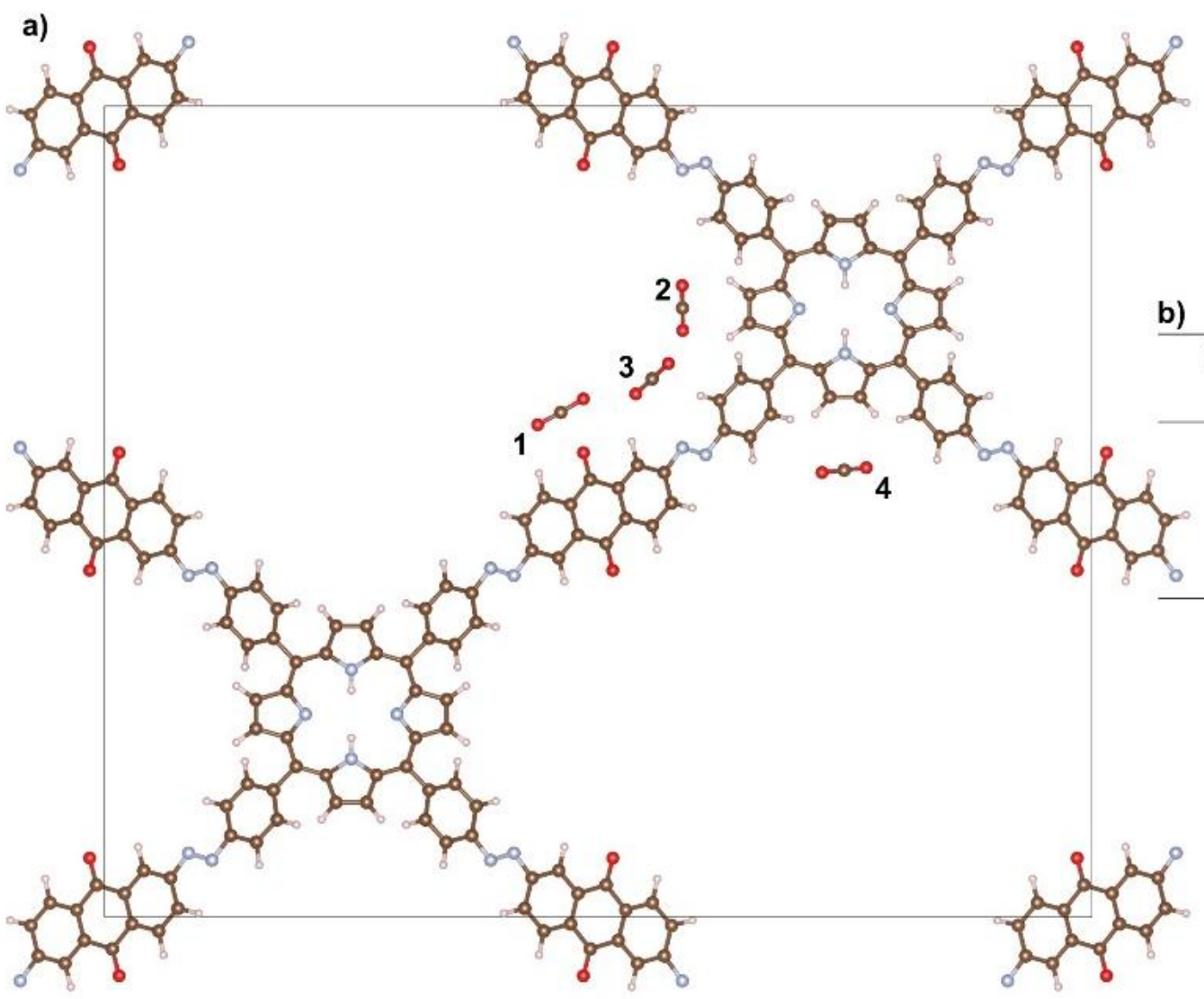

b)

CO_2···APP-AQ complex	Relative energy (kJ mol^{-1})	Interaction energy (kJ mol^{-1})
1	0.00	–27.13
2	0.42	–18.31
3	0.59	–18.68
4	3.39	–16.20

Figure S22. a) Overlay of optimized geometries (PBE-D3/pob-TZVP-rev2) for the selected CO_2···APP-AQ complexes, illustrating different CO_2 binding positions within the framework. All complexes are superimposed to highlight structural variations in CO_2 adsorption sites. b) Table summarizing the relative energies (in kJ mol^{-1}) of the complexes along with BSSE-corrected interaction energies between a single CO_2 and the APP-AQ framework.

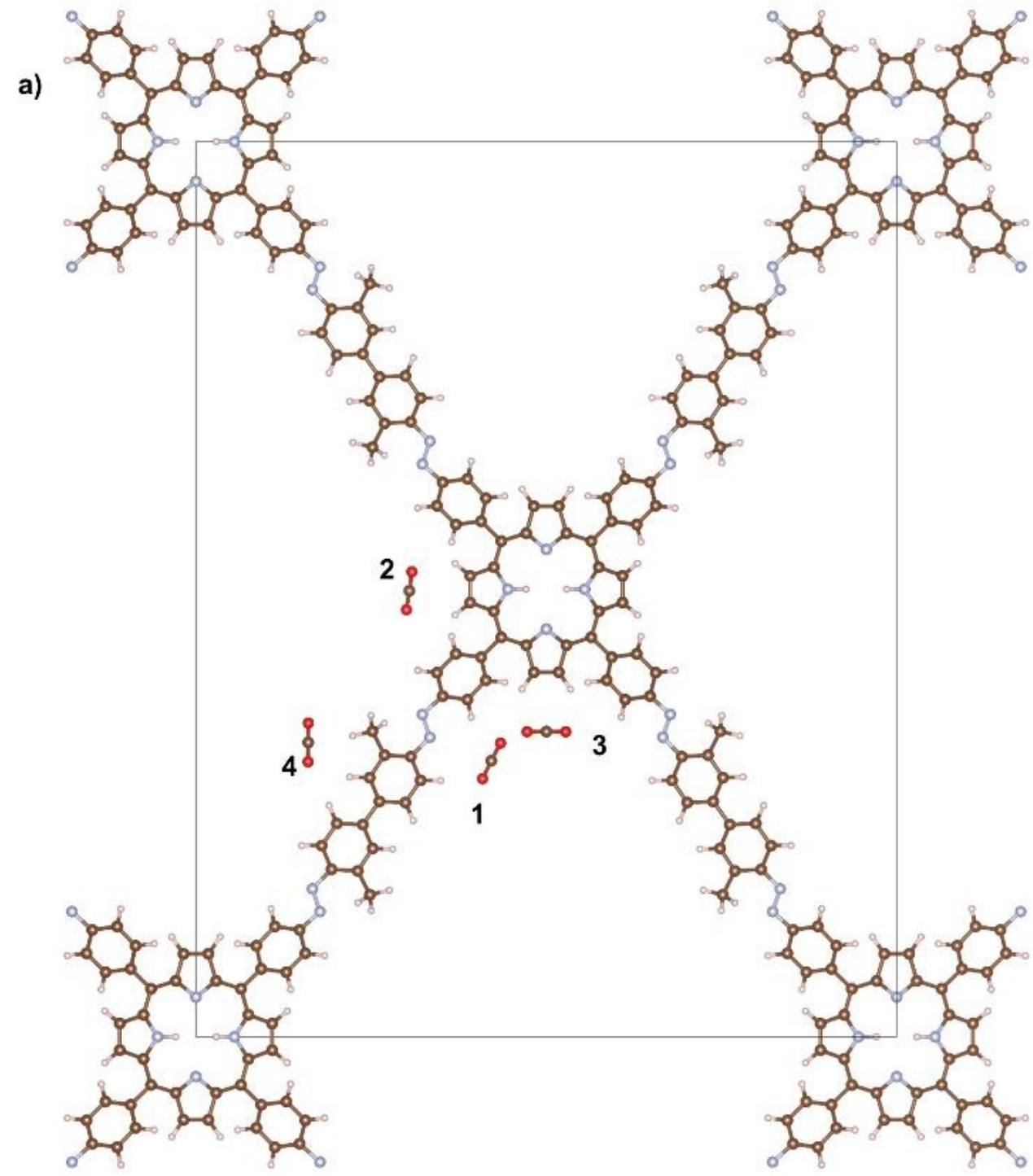

b)

$CO_2\cdots$APP-BP-Me complex	Relative energy (kJ mol^{-1})	Interaction energy (kJ mol^{-1})
1	0.00	–19.15
2	0.76	–17.66
3	0.81	–18.15
4	9.08	–11.47

Figure S23. a) Overlay of optimized geometries (PBE-D3/pob-TZVP-rev2) for the selected $CO_2\cdots$APP-BP-Me complexes, illustrating different CO_2 binding positions within the framework. All complexes are superimposed to highlight structural variations in CO_2 adsorption sites. b) Table summarizing the relative energies (in kJ mol^{-1}) of the complexes along with BSSE-corrected interaction energies between a single CO_2 and the APP-BP-Me framework.

2.6. Fractional atomic coordinates and unit cell parameters

Table S7. Fractional atomic coordinates for the unit cell of APP-BP-OH.

APP-BP-OH, Space group : *P m n a*			
a = 43.895554 Å, b=3.706386 Å, c=38.517643 Å			
$\alpha = \beta = \gamma = 90^{\circ}$			
Atom	x	y	z
N1	0.171992	-0.065261	0.158394
N2	0.046829	-0.500000	0.000000
N3	0.000000	0.312796	0.051350
N4	0.145587	0.079944	0.166599
C5	0.065821	0.400303	0.026932
C6	0.056486	0.280363	0.060171
C7	0.097560	0.441172	0.016670
C8	0.025893	0.226959	0.070114
C9	0.124667	0.116611	0.139353
C10	0.192357	-0.094558	0.185622
C11	0.237860	-0.145013	0.236836
C12	0.221475	-0.233434	0.177439
C13	0.208852	-0.021142	0.245740
C14	0.129146	-0.023186	0.105816
C15	0.106867	0.021740	0.080792
C16	0.079777	0.215493	0.087377
C17	0.097506	0.300515	0.146885
C18	0.075789	0.352439	0.121228
C19	0.186007	0.013195	0.220625
C20	0.244001	-0.254054	0.202257
C21	0.015604	0.065054	0.101720
H22	0.000000	0.429765	0.027251
H23	0.030392	-0.042010	0.121693
H24	0.117478	0.386703	0.032422
H25	0.149736	-0.178069	0.100571
H26	0.109637	-0.104831	0.055505
H27	0.055541	-0.489329	0.127029
H28	0.266407	-0.361995	0.195829
H29	0.094331	0.412656	0.172831
H30	0.225247	-0.320671	0.150732
H31	0.203886	0.071054	0.272026
O32	-0.341178	0.152840	0.270765
H33	-0.146820	0.158366	0.205478

Table S8. Fractional atomic coordinates for the unit cell of APP-AQ.

APP-AQ, Space group : *P* 21/m			
a = 36.4450 Å, b= 44.3738 Å, c= 3.7799 Å			
$\alpha = \gamma = 90^{\circ}$, $\beta = 100.32^{\circ}$			
Atom	x	y	z
O1	0.927408	0.985091	0.261071
N2	0.805062	0.250000	0.159596
N3	0.749993	0.203667	0.249907
N4	0.921173	0.085238	0.857467
N5	0.930091	0.108886	0.056477
N6	0.578817	0.085252	0.642723
N7	0.569902	0.108883	0.443396
C8	0.778813	0.184870	0.199508
C9	0.814392	0.194139	0.140922
C10	0.767834	0.153454	0.221100
C11	0.825055	0.224398	0.106419
C12	0.960584	0.991938	0.368995
C13	0.863109	0.123482	0.888828
C14	0.835968	0.144875	0.908337
C15	0.899632	0.127996	0.076781
C16	0.907741	0.153912	0.289181
C17	0.880069	0.174923	0.312106
C18	0.843479	0.171168	0.119478
C19	0.685588	0.194139	0.358713
C20	0.721172	0.184871	0.300264
C21	0.732152	0.153454	0.278759
C22	0.641255	0.234562	0.499594
C23	0.674914	0.224397	0.393066
C24	0.636881	0.123485	0.611032
C25	0.664019	0.144880	0.591482
C26	0.600359	0.127993	0.423019
C27	0.592250	0.153898	0.210421
C28	0.619922	0.174906	0.187382
C29	0.656507	0.171164	0.380180
C30	0.548112	0.066923	0.681879
C31	0.510473	0.074392	0.552751
C32	0.557120	0.039967	0.869736
C33	0.482541	0.054968	0.613051
C34	0.528971	0.020389	0.931209
C35	0.491338	0.027951	0.803591
C36	0.539416	0.991964	0.131516
C37	0.951881	0.066913	0.818282
C38	0.989520	0.074403	0.946984
C39	0.942875	0.039943	0.630710
C40	0.017455	0.054990	0.886504
C41	0.971027	0.020376	0.569044

C42	0.991339	0.972039	0.303802
O43	0.427408	0.014860	0.760067
C44	0.858695	0.234562	0.999510
N45	0.694914	0.250000	0.340059
H46	0.779326	0.250000	0.232874
H47	0.879842	0.219897	0.925964
H48	0.936021	0.156712	0.439363
H49	0.784746	0.133768	0.195310
H50	0.715241	0.133769	0.304564
H51	0.620078	0.219903	0.572681
H52	0.642631	0.103706	0.783033
H53	0.691651	0.142253	0.754069
H54	0.563973	0.156690	0.060144
H55	0.613583	0.194386	0.011927
H56	0.504284	0.095424	0.405333
H57	0.586215	0.034134	0.968643
H58	0.453113	0.059848	0.515532
H59	0.995708	0.095446	0.094155
H60	0.913780	0.034093	0.532127
H61	0.046884	0.059887	0.983696
H62	0.808335	0.142240	0.745805
H63	0.886420	0.194417	0.487316
H64	0.857358	0.103694	0.716979
H65	0.720663	0.250000	0.267067

Table S9. Fractional atomic coordinates for the unit cell of APP-BP-Me.

APP-BP-Me, Space group : *P m n a*			
a = 46.1459 Å, b= 36.1217 Å, c= 3.7258 Å			
$\alpha = \beta = \gamma = 90^{\circ}$			
Atom	x	y	z
N1	0.165329	0.833794	0.023628
N2	0.044476	0.000000	0.500000
N3	0.000000	0.945244	0.312287
N4	0.139870	0.823061	0.131440
C5	0.062550	0.971286	0.400860
C6	0.053695	0.935829	0.281716
C7	0.092747	0.982218	0.441689
C8	0.024624	0.925160	0.228630
C9	0.119389	0.852053	0.145072
C10	0.187083	0.806619	0.015651
C11	0.237041	0.761805	0.982736
C12	0.213585	0.820521	0.883848
C13	0.210472	0.747861	0.107680
C14	0.123304	0.887416	0.995092
C15	0.101728	0.913793	0.027923
C16	0.075943	0.906832	0.220693
C17	0.093305	0.843907	0.321447
C18	0.072265	0.870995	0.363377
C19	0.158692	0.751779	0.289769
C20	0.185343	0.769143	0.133316
C21	0.238268	0.798873	0.869578
C22	0.014846	0.891301	0.070488
H23	0.000000	0.971043	0.426565
H24	0.028952	0.869874	0.967060
H25	0.111674	0.965384	0.387461
H26	0.163803	0.723963	0.391987
H27	0.143049	0.892944	0.843825
H28	0.104105	0.940460	0.895164
H29	0.052874	0.864939	0.518862
H30	0.258487	0.810066	0.763429
H31	0.149773	0.768837	0.507354
H32	0.141188	0.749727	0.090196
H33	0.090675	0.816457	0.439133
H34	0.213634	0.849233	0.793170
H35	0.209764	0.719311	0.204878

Table S10. Fractional atomic coordinates for the unit cell of APP-Ph-Me.

APP-Ph-Me, Space group : *C* 2/m			
a = 32.6650 Å, b=36.4876 Å, c=3.8275 Å			
$\alpha = \gamma = 90^{\circ}$, $\beta = 94.01^{\circ}$			
Atom	x	y	z
N1	-0.060819	0.000000	0.284373
N2	0.000000	0.056302	-0.500000
N3	-0.186969	0.201109	-0.296284
N4	-0.197259	0.172291	-0.121546
C5	-0.019626	0.117361	0.429055
C6	-0.070878	0.067912	0.240712
C7	-0.031698	0.079155	0.382013
C8	-0.119706	0.018781	-0.002779
C9	-0.082740	0.031127	0.183174
C10	-0.219487	0.224863	-0.395717
C11	-0.208532	0.257032	0.423035
C12	-0.123895	0.153569	-0.133779
C13	-0.094214	0.127481	-0.047449
C14	-0.164098	0.148677	-0.031685
C15	-0.173174	0.117491	0.163516
C16	-0.142943	0.091806	0.253647
C17	-0.102688	0.095902	0.148256
C18	-0.260488	0.218385	-0.322941
C19	-0.164997	0.263772	0.338416
H20	-0.038336	0.141233	0.363047
H21	-0.032535	0.000000	0.418285
H22	-0.142892	0.036650	-0.126296
H23	-0.117474	0.177197	-0.295419
H24	-0.063810	0.130239	-0.144120
H25	-0.150023	0.068406	0.415540
H26	-0.267098	0.193509	-0.179288
H27	-0.154280	0.241593	0.172201
H28	-0.144101	0.264450	-0.423599
H29	-0.162620	0.289905	0.198606
H30	-0.204219	0.114556	0.247986

References

(1) Zhao, M.; Zhu, S.; Yang, X.; Wang, Y.; Zhou, X.; Xie, X. A Porphyrinic Donor–Acceptor Conjugated Porous Polymer as Highly Efficient Photocatalyst for PET–RAFT Polymerization. *Macromol. Rapid Commun.* **2022**, *43* (18). https://doi.org/10.1002/marc.202200173.

(2) Panić, B.; Frey, T.; Borovina, M.; Ištoković, P.; Kodrin, I.; Biljan, I. Synergistic Experimental and Computational Investigation of Azo-Linked Porphyrin-Based Porous Organic Polymers for CO_2 Capture. *RSC Adv.* **2025**, *15* (18), 13774–13785. https://doi.org/10.1039/d4ra08113g.
